# Recent advances of self-assembled nanoparticles in the diagnosis and treatment of atherosclerosis

**DOI:** 10.7150/thno.100388

**Published:** 2024-11-04

**Authors:** Tuersun Aili, Jia-bin Zong, Yi-fan Zhou, Yu-xiao Liu, Xiang-liang Yang, Bo Hu, Jie-hong Wu

**Affiliations:** 1 Department of Neurology, Union Hospital, Tongji Medical College, Huazhong University of Science and Technology, Wuhan 430022, China.; 2 National Engineering Research Center for Nanomedicine, College of Life Science and Technology, Huazhong University of Science and Technology, Wuhan 430074, China.

**Keywords:** self-assembled nanoparticles, nanoparticles, atherosclerosis, diagnosis, treatment

## Abstract

Atherosclerosis remains a significant global health challenge, with its related conditions as the leading cause of death, underscoring the urgent need for enhanced diagnostic and therapeutic approaches. Recently, self-assembled nanoparticles (SANPs) have shown remarkable promise in treating atherosclerosis, attributed to their superior bioavailability, biodegradability, biocompatibility, and ease of functional modification. Numerous SANP variants, such as DNA origami, metal-organic frameworks (MOFs), nanozymes, peptide-based nanoparticles, and self-assembled prodrug nanoparticles, have been engineered, extending their utility in targeted drug delivery and imaging. Advances in fabrication technologies, including microfluidic techniques, allow for precise and scalable SANP production, while innovative nanoparticle designs—such as stimuli-responsive and carrier-free variants—enhance pharmacokinetic properties. The deployment of SANPs in atherosclerosis has introduced a range of diagnostic and therapeutic solutions, from non-invasive imaging and stimuli-responsive drug delivery to vaccination, theranostics, and biosensing. This review consolidates the recent progress in SANP applications for atherosclerosis, emphasizing their transformative potential in disease management.

## 1. Introduction

Atherosclerosis is a chronic inflammatory condition characterized by the accumulation of lipids and inflammatory agents within arterial walls, leading to plaque formation and reduced blood flow. Its severe complications, including myocardial infarction and stroke, are among the top causes of mortality worldwide 1-3. Traditional imaging methods like computed tomography (CT) and magnetic resonance imaging (MRI) offer detailed insights into the arterial wall's morphological aspects, such as volume and thickness. However, these techniques are limited in their capacity to detect early-stage atherosclerosis and assess plaque vulnerability and progression accurately 4-6. Conventional therapies, such as statins, focus primarily on lowering circulating lipid levels to decelerate disease progression. Yet, their efficacy is constrained, with prolonged statin use linked to side effects like hepatotoxicity, muscle pain, and an increased risk of new-onset type 2 diabetes mellitus 7, 8. This highlights the pressing need for innovative strategies in early diagnosis and effective treatment.

Nanoparticle (NP)-based technologies offer promising avenues for the targeted imaging and treatment of atherosclerosis by improving drug delivery and stability, reducing toxicity, and prolonging circulation time 9, 10. NPs can direct radiotracers and contrast agents to specific molecules within atherosclerotic plaques, enabling more precise imaging and plaque detection 9, 11. Additionally, they facilitate selective delivery of therapeutic agents to affected cells, slowing disease progression. However, challenges persist in optimizing nanoparticle properties for clinical use, particularly concerning scalability and efficient production 12, 13.

Self-assembled nanoparticles (SANPs) are formed through a spontaneous self-assembly process driven by non-covalent interactions, which result in the organization of components at the nanoscale. These nanocarriers are favored for their ease of fabrication, structural versatility, and exceptional stability in aqueous environments 13, 14. As a result, self-assembly is considered a powerful approach for creating nanoscale biomaterial delivery systems, making SANPs especially suitable for addressing the complexities of atherosclerosis treatment.

**Figure 1:**
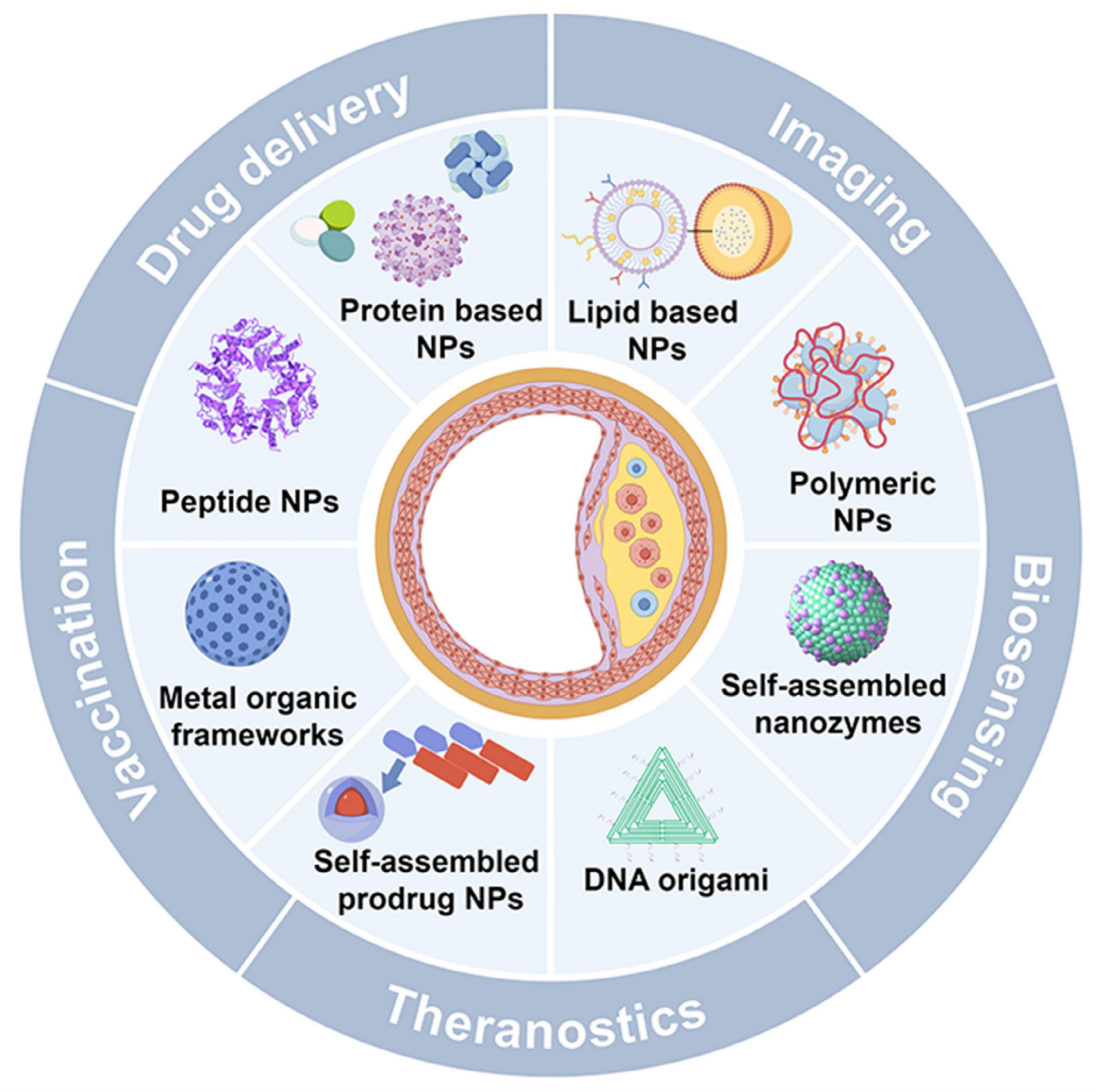
Schematic for SANPs based strategies for diagnosis and treatment of atherosclerosis. Created by Figdraw.

To further illustrate the advantages of SANPs over other nanoparticle systems, Table 1 outlines the key distinctions in synthesis, structural properties, material composition, surface functionalization, and clinical applicability 13-17. This review delves into the role of SANPs in diagnosing and treating atherosclerosis (Figure 1), emphasizing the latest advances, clinical progress, and future challenges.

## 2. Pathophysiology of atherosclerosis

Atherosclerosis is a lipoprotein-driven inflammatory disorder, defined by the development of plaques within arterial walls. Pro-inflammatory cytokines and related stimuli prompt endothelial cells to express adhesion molecules and cytokines, facilitating the recruitment and transmigration of monocytes into the intima 10. Once in the vessel wall, monocytes differentiate into macrophages, which can adopt either an inflammatory (M1) or anti-inflammatory (M2) phenotype. M1 macrophages absorb cholesterol, transforming into foam cells. When unable to process excess cholesterol, these cells undergo cytotoxicity and apoptosis, contributing to necrotic core formation and exacerbating inflammation 9. As the condition advances, smooth muscle cells (SMCs) in the media transition from a contractile to a proliferative phenotype, migrating into the intima where they produce collagen to form a protective fibrous cap over plaques. However, SMCs may also transform into foam cells, undergo apoptosis, and further contribute to necrotic core formation and inflammation 2, 10. Elevated lipid levels and ongoing inflammation ultimately create vulnerable plaques characterized by large necrotic cores and thin fibrous caps, heightening the risk of plaque rupture, thrombosis, and sudden cardiac death 9. Early detection and intervention are essential to mitigate these risks. Advances in nanotechnology, particularly SANPs, offer innovative solutions for the precise diagnosis and treatment of atherosclerosis.

## 3. Overview of self-assembled nanoparticles for atherosclerosis

### 3.1. Self-assembly principles and nanoparticle types

Self-assembly is a thermodynamically driven process in which smaller building blocks—such as polymers, lipids, or proteins—spontaneously form well-defined, ordered structures. This occurs through the minimization of free energy and relies on non-covalent interactions such as electrostatic forces, hydrophobic interactions, van der Waals forces, hydrogen bonding, π-π stacking, and metal coordination 14, 16. These interactions give rise to stable, intricate structures, commonly found in biological systems like DNA helices and protein folding 15, 16. Inspired by these natural processes, self-assembly principles have been leveraged to design sophisticated nanomaterials, particularly SANPs. These nanoparticles form spontaneously from small molecules without requiring external forces or complex fabrication techniques. Their size, shape, and surface properties can be finely tuned, and they exhibit enhanced bioavailability, biodegradability, biocompatibility, and ease of modification, making them highly promising for clinical applications 15-17. SANPs for atherosclerosis include lipid-based, protein-based, polymeric, peptide nanoparticles, and metal-organic frameworks (MOFs), with newer developments encompassing self-assembled prodrug nanoparticles, nanozymes, and DNA origami, each with distinct properties suited to specific uses.

Lipid-based nanoparticles, such as liposomes and lipid nanoparticles (LNPs), are renowned for their biocompatibility and their ability to encapsulate both hydrophilic and hydrophobic drugs 18. Protein-based nanoparticles offer the advantages of biodegradability and biocompatibility, making them ideal for therapeutic applications 19, 20. Peptide nanoparticles, composed of self-assembling peptides, are highly tunable and biocompatible, making them excellent candidates for drug delivery and molecular recognition 21, 22. Polymeric nanoparticles, made from biodegradable polymers, are versatile and can be engineered for controlled drug release 23, 24. MOFs, with their crystalline structure composed of metal ions and organic ligands, are noted for high porosity and tunable architecture, ideal for drug delivery and catalysis 25. Additionally, self-assembled prodrug nanoparticles, which form without the need for external carriers, reduce systemic toxicity and enhance drug-loading efficiency, offering novel pathways for precision medicine 26. Beyond drug delivery, self-assembled nanomaterials extend into other functional domains. For instance, nanozymes mimic enzymatic activity, offering stability and customizable catalytic properties, making them useful in therapeutic catalysis 27, 28. DNA origami, which uses DNA strands to create intricate nanostructures, allows for targeted drug delivery, biosensing, and other biomedical applications 29, 30. Table 2 provides a detailed comparison of these SANPs, summarizing their key features, advantages, disadvantages, and preparation methods, while also highlighting their potential applications in atherosclerosis management.

### 3.2. Preparation methods and design strategies

**Figure 2:**
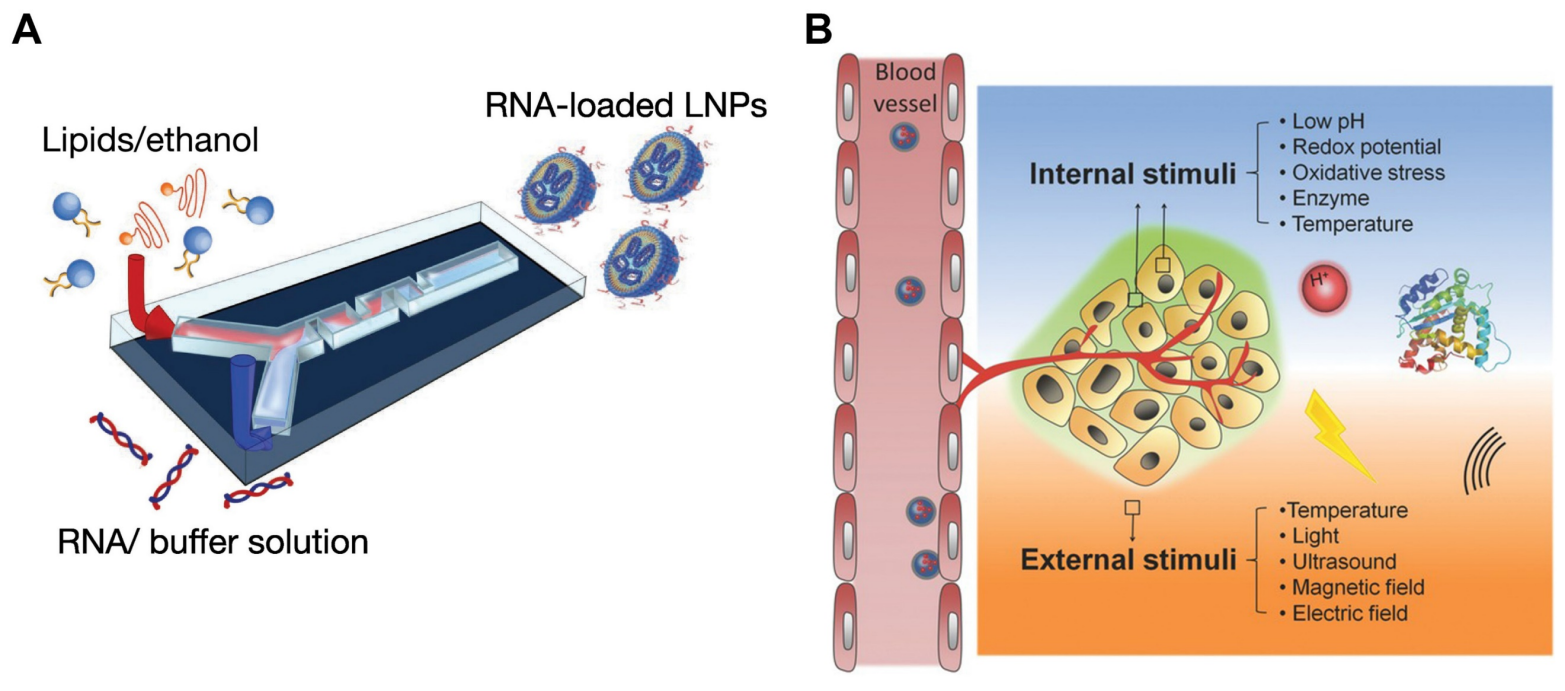
Schematic illustrations for new preparation methods and design strategies for developing self-assembled nanoparticles. A. Schematic illustration for preparing RNA-loaded lipid nanoparticles using microfluidic device. Reproduced with permission from 35. Copyright 2022, Elsevier. B. Schematic illustrations of stimuli responsive nanoparticles and various stimuli employed for controlled drug release. Reproduced with permission from 36. Copyright 2014, John Wiley and Sons.

The preparation methods for self-assembled nanoparticles have advanced considerably, with various techniques developed to regulate their size, shape, and functionality. Traditional methods such as solvent evaporation, nanoprecipitation, and emulsification, though effective, often lack the precision necessary for fine-tuning nanoparticle properties 31, 32. The introduction of microfluidic technology has revolutionized nanoparticle fabrication, offering distinct advantages over conventional approaches. Microfluidics enables precise control over fluid dynamics at the microscale, producing nanoparticles with uniform size and morphology. This technology facilitates rapid mixing of reagents, ensuring high reproducibility and scalability in nanoparticle production (Figure 2A). Additionally, microfluidic devices can integrate multiple stages of nanoparticle synthesis within a single platform, increasing efficiency and minimizing the need for extensive purification steps. The precise control of reaction conditions in microfluidics also paves the way for creating more complex, multifunctional nanoparticles 32-34.

The evolution of self-assembled nanoparticles has led to increasingly sophisticated systems with enhanced functionality. Multifunctional nanoparticles are being engineered to carry multiple therapeutic agents or to combine therapeutic and diagnostic capabilities (theranostics) in a single platform 37. Active targeting represents a significant advancement, whereby nanoparticle surfaces are modified with targeting ligands such as antibodies, peptides, or aptamers, enabling selective binding to specific cells or tissues 38, 39. This targeted approach enhances drug accumulation at the intended site, reduces off-target effects, and improves therapeutic efficacy. Additionally, cell membrane coating technology employs natural cell membranes to cloak nanoparticles, enhancing biocompatibility, prolonging circulation time, and minimizing immune clearance. This method takes advantage of the natural targeting abilities and immune evasion properties of cell membranes, significantly improving the *in vivo* stability and targeting efficiency of nanoparticles 40, 41. Another strategy, cell hitchhiking, involves attaching nanoparticles to the surface of live cells like macrophages, harnessing the cells' migratory and targeting capabilities to further enhance nanoparticle delivery efficiency 42.

Moreover, stimuli-responsive nanoparticles are engineered to react to specific triggers, such as pH changes, reactive oxygen species (ROS), or the presence of particular enzymes, enabling controlled and site-specific drug release (Figure 2B). These systems offer precise drug delivery control, minimizing side effects and enhancing therapeutic outcomes 43-46. Recent innovations also include carrier-free nanoparticles, where therapeutic agents themselves form the nanoparticle structure through self-assembly, eliminating the need for additional carriers and reducing potential toxicity 47. These developments in self-assembled nanoparticles promise significant advances in nanomedicine, offering more effective and personalized treatment strategies.

## 4. SANPs for atherosclerosis imaging

Traditional imaging methods for atherosclerosis, such as coronary angiography, are invasive and limited in their ability to fully characterize plaque structure. While non-invasive techniques like MRI and positron emission tomography (PET) lower patient risk by detecting inflammation and calcification, they remain inadequate for early plaque identification and vulnerability assessment 4, 5. Nanoparticles, due to their small size, can infiltrate plaque interiors, offering deeper insights into disease mechanisms 57. SANPs further enhance imaging resolution through superior biocompatibility, stability, and targeting capabilities. Recent studies have integrated SANPs with MRI, photoacoustic imaging (PAI), and fluorescence imaging, enabling improved detection and characterization of high-risk plaques.

In MRI applications, nanoparticles function as contrast agents, enhancing the visualization of soft tissues, which is essential for detecting plaques and cellular components in atherosclerosis. SANPs provide advantages such as easy surface modification and high biocompatibility, increasing both targeting efficiency and safety. One study developed a composite nanoparticle system composed of low molecular weight fucoidan (LMWF) and protamine peptide (TPP1880), loaded with the MRI contrast agent Gd-DTPA. LMWF specifically targets P-selectin at inflammation sites and plaques, while TPP1880, a cell-penetrating peptide, facilitates Gd-DTPA delivery, improving imaging performance. This system demonstrated enhanced targeting of P-selectin-expressing cells, greater T1 relaxivity, and reduced cytotoxicity, indicating superior safety and imaging efficacy 58.

**Figure 3:**
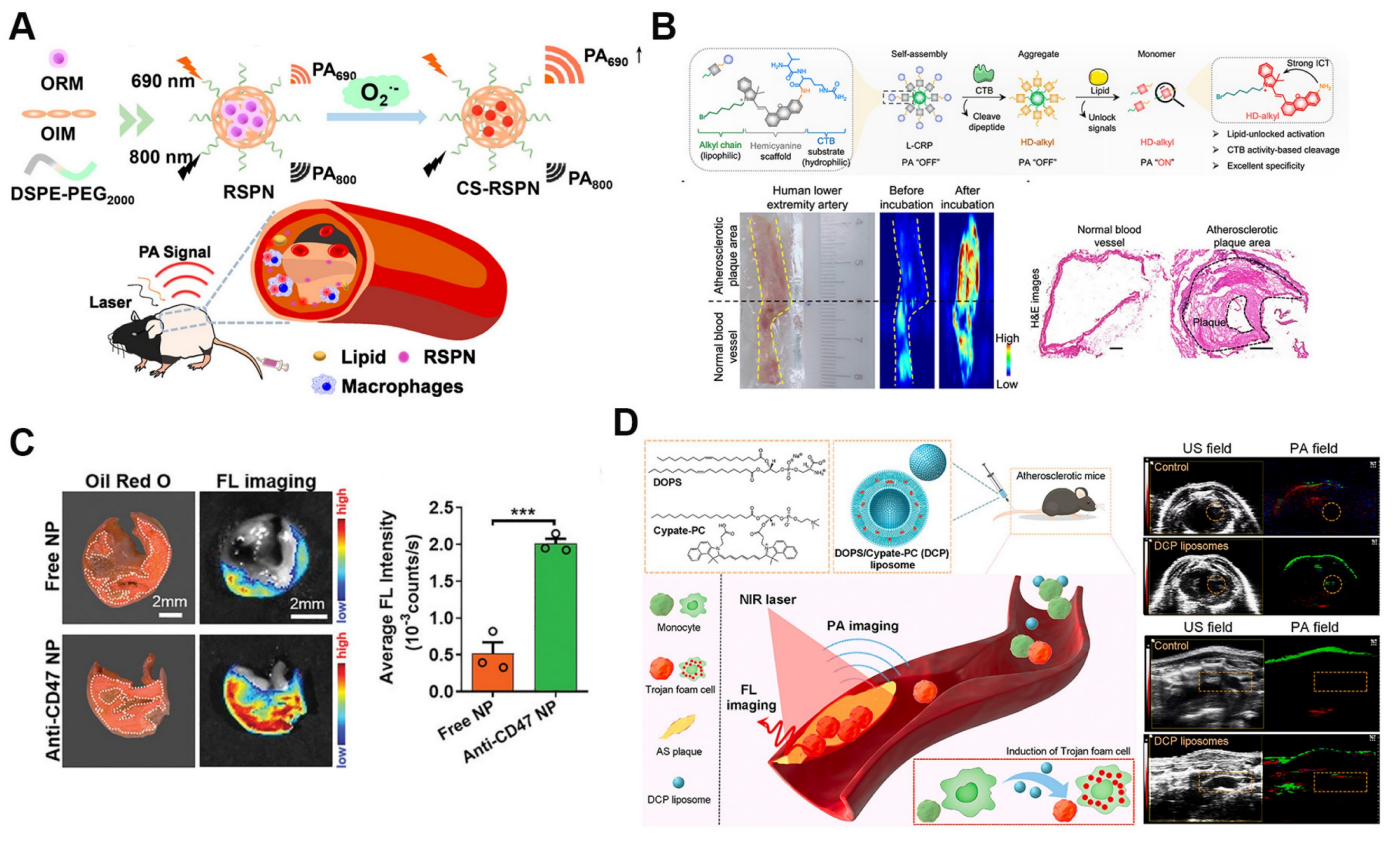
Self-assembled nanoparticles for atherosclerosis non-invasive imaging. A. Schematic illustration of ratiometric photoacoustic semiconducting polymer nanoparticles (RSPNs) for *in vivo* imaging of atherosclerotic plaques. RSPNs respond to superoxide anions (O₂⁻), enhancing the photoacoustic signal at 690 nm with 800 nm as a reference, enabling specific detection of oxidative stress within plaques. Reproduced with permission from 60. Copyright 2021, American Chemical Society. B. Design and application of lipid-unlocking CTB-reactive probe (L-CRP) for specific imaging of atherosclerotic plaques. The L-CRP activates in the presence of CTB and lipids, providing enhanced photoacoustic imaging signals and distinguishing atherosclerotic lesions from normal vessels. Reproduced with permission from 61. Copyright 2022, American Chemical Society. C. Fluorescence imaging reveals a fourfold greater accumulation of anti-CD47 NPs in the lipid core of human carotid plaques compared to free NPs, confirming their enhanced targeted binding efficacy. Reproduced with permission 62. Copyright 2020, John Wiley and Sons. D. Schematic for the plaque-targeted imaging with DCP liposomes and *in vivo* targeted imaging ability of plaque by PAI and FI. The successful recognition of aortic arch by photoacoustic imaging in which the green PA signal appeared in the left thoracic cavity was confirmed by ultrasonic imaging. Reproduced with permission 63. Copyright 2022, Elsevier.

PAI is a cutting-edge technique for diagnosing atherosclerosis, offering high resolution, deep tissue penetration, and superior contrast. Compared to methods like CT, PAI more effectively reveals oxidative stress and inflammatory markers within plaques, providing key information about plaque vulnerability 59. SANPs can be engineered to respond to biochemical signals, such as pH and redox changes within plaques, allowing selective activation in diseased tissues and enhancing PAI contrast and specificity. For example, ratiometric photoacoustic semiconducting polymer nanoparticles (RSPNs) offer a highly specific approach for *in vivo* plaque imaging (Figure 3A). RSPNs are self-assembled from O_2_^-^-responsive and O_2_^-^-insensitive semiconducting polymers combined with an amphiphilic polymer (DSPE-PEG_2000_) using nanoprecipitation. These nanoparticles react with superoxide anions (O_2_^-^), amplifying the photoacoustic signal at 690 nm, while 800 nm serves as an internal reference. The signal ratio at these wavelengths allows for precise measurement of O_2_^-^ levels in plaques, indicating oxidative stress in vulnerable plaques 60. Another study introduced a lipid-unlocking CTB-reactive probe (L-CRP) designed for imaging CTB activity in plaques. L-CRP consists of a CTB-responsive dipeptide, a lipophilic alkyl chain, and an encapsulated hemicyanine scaffold (Figure 3B). This probe generates a photoacoustic signal only in the presence of both CTB and lipids, ensuring high specificity and reducing the likelihood of false activation. As a result, it produces stronger PA signals within plaques, with deep tissue penetration exceeding 1.0 cm. The study found that the PA signal of L-CRP is inversely correlated with fibrous cap thickness, enabling differentiation between atherosclerotic and healthy mice and facilitating risk stratification. Additionally, L-CRP effectively distinguished atherosclerotic plaques from normal vessels in human arterial tissue, underscoring its clinical potential 61.

Fluorescence imaging (FI) plays a pivotal role in studying atherosclerosis by enabling real-time visualization of lipid droplets (LDs) at the cellular level. However, its *in vivo* application has been hindered by the hydrophobicity, low signal-to-noise ratio (SNR), and poor specificity of traditional LD-targeting probes. To overcome these limitations, a recent study introduced a self-assembled nanoparticle system incorporating a fluorescent probe, MeOND, within a ROS-responsive platform. These nanoparticles enhance near-infrared emission in low-polarity environments like LDs while remaining stable under physiological conditions and degrading in response to elevated ROS levels. This design significantly improves real-time imaging specificity and resolution of LDs in atherosclerotic plaques, advancing non-invasive diagnostic capabilities 64. Another innovative study developed a smart-responsive nanoparticle system, HA@PCFT, designed for lipid-specific imaging of vulnerable atherosclerotic plaques. This system employs a fluorescent probe, FC-TPA, which activates fluorescence through hydrogen bonding, thereby reducing background interference and improving imaging accuracy. FC-TPA is encapsulated in cyclodextrin (CD), modified with a phosphatidylserine-targeting peptide (PTP), and coated with hyaluronic acid (HA) for dual targeting. HA binds to the CD44 receptor, while PTP targets phosphatidylserine on apoptotic foam cells, allowing for precise plaque accumulation. Upon ROS stimulation, FC-TPA is released, binds to lipids, and emits green fluorescence, providing high-resolution visualization of plaque lipid content. *In vitro* and *in vivo* studies confirmed the efficacy of HA@PCFT in delivering high-resolution plaque imaging, with potential for early diagnosis and vulnerability assessment, which could mitigate cardiovascular mortality through timely intervention 65.

Recent advances in fluorescence imaging for atherosclerosis have focused on reducing noise and enhancing specificity while improving luminophores' brightness and photostability for more accurate lesion detection at earlier stages. Aggregation-induced emission (AIE), where molecules emit stronger fluorescence when aggregated in high concentrations or confined environments like biological tissues, represents a promising strategy 66. AIE luminophores, with their high brightness, photostability, and low background noise, are ideal for imaging applications. An innovative study introduced a self-assembled AIE nanoprobe, TPE-T-RCN, specifically designed for near-infrared fluorescence imaging of atherosclerotic plaques. This nanoprobe offers high brightness and specificity by targeting the upregulated CD47 molecule in plaques. TPE-T-RCN, modified with rhodanine to enhance its photophysical properties, achieves superior molar extinction coefficient, photoluminescence quantum yield, and red-shifted spectra compared to other compounds tested. Formulated with an amphiphilic copolymer and anti-CD47 antibodies, it enables early, high-contrast plaque detection, outperforming conventional imaging methods. This nanoprobe also provides a faster alternative to staining, efficiently detecting human carotid plaques, and holds promise as a screening tool for anti-atherosclerosis drugs (Figure 3C) 62.

While single-mode imaging techniques, such as fluorescence or photoacoustic imaging, offer valuable insights, they often suffer from limitations in sensitivity, specificity, or depth penetration. Multimodal imaging, which integrates multiple imaging modalities, leverages their complementary strengths, significantly improving diagnostic accuracy and allowing for more thorough characterization of atherosclerotic plaques 67. For instance, innovative DCP liposomes utilize a LD hitchhiking method to generate "Trojan foam cells" that facilitate fluorescence and photoacoustic imaging of atherosclerotic plaques. Comprising the LD-inducing dioleoylphosphatidylserine (DOPS) and the molecular probe Cypate-PC, these liposomes effectively trigger lipid droplet formation within macrophages. Encapsulated within the foam cells, the imaging probe is effectively targeted to plaque locations. The LDs' hydrophobic nature significantly improves the probe's light absorption, enhancing both fluorescence and photoacoustic imaging signals (Figure 3D). This dual-functional system allows for *in vivo* photoacoustic detection of atherosclerotic plaques, providing a powerful tool for visualizing plaques 63. Moreover, ^89^Zr-radiolabeled liposomes used in PET/CT facilitate long-term tracking of liposomes *in vivo*, offering new opportunities for assessing atherosclerotic burden over time 68.

In summary, SANPs show immense potential for improving the diagnosis of atherosclerosis by enhancing imaging resolution and targeting precision. Table 3 outlines notable examples of SANPs used in non-invasive imaging. Looking ahead, advancements in multifunctional nanoplatforms and their integration into multimodal imaging techniques are poised to revolutionize atherosclerosis management, enabling more precise, real-time clinical applications.

## 5. SANPs in atherosclerosis treatment

### 5.1. SANPs in atherosclerosis drug delivery

The introduction of nanocarriers has revolutionized targeted drug delivery, offering reduced systemic toxicity and extended circulation times. However, challenges such as poor biocompatibility and high production costs persist 12. Self-assembled nanodrug delivery systems address these issues by enhancing drug stability, targeting specificity, and controlled release while providing good biocompatibility and longer circulation 13, 14. These systems, which include lipid-based nanoparticles, protein-based nanoparticles, peptide nanoparticles, polymeric nanoparticles, and self-assembled prodrug nanoparticles, have shown significant efficacy in treating atherosclerosis. Recent developments, such as DNA origami and carrier-free nanodrugs, also demonstrate substantial promise in modulating therapeutic processes for atherosclerosis management.

#### 5.1.1. Lipid based nanoparticles for atherosclerosis drug delivery

Lipid-based nanoparticles have emerged as a leading platform for atherosclerosis treatment due to their excellent biocompatibility, ability to encapsulate both hydrophilic and hydrophobic drugs, and capacity for targeted delivery. Among them, liposomes are widely used as nanocarriers for drugs such as simvastatin and rapamycin (RAP), with proven efficacy in treating atherosclerosis 69. Recently, Chong *et al.* developed an injectable liposomal formulation of docosahexaenoic acid (DHA), known for its strong anti-inflammatory and antioxidant properties, specifically targeting atherosclerotic plaques. This formulation not only protects DHA from degradation but also enhances its concentration within atherosclerotic lesions. Upon intravenous injection, these DHA liposomes selectively accumulate in lesional macrophages, promoting M2 macrophage polarization and reducing atherosclerosis progression 70. In another development, researchers designed nano-sponge-like liposomes (Rb1-LPs) by incorporating ginsenosides Rb1 into soy phospholipid bilayers, further modifying them with annexin V to form AnxV-Rb1-LPs. *In vitro* studies revealed that AnxV-Rb1-LPs solubilized cholesterol crystals, reducing their accumulation. When administered intravenously, these liposomes specifically targeted atherosclerotic plaques, effectively eliminating intra- and extracellular cholesterol crystals, offering a novel approach for cholesterol crystal clearance within plaques 71.

Biomimetic liposomes, coated with cell membranes, offer enhanced biocompatibility, reduced immunogenicity, and improved targeting due to their resemblance to natural cell membranes. For example, macrophage membrane-coated liposomes (MM@Lips-SHP1i) encapsulating an SHP1 inhibitor (SHP1i) have shown significant potential in atherosclerosis treatment by targeting the CD47 signaling pathway. These nanoparticles evade immune detection, extend circulation time, and preferentially accumulate in atherosclerotic plaques. By competing with macrophages for oxidized LDL (ox-LDL) binding, they reduce foam cell formation and inhibit pro-inflammatory cytokine expression. Moreover, the SHP1 inhibitor disrupts the CD47-SIRPα signaling pathway, promoting macrophage efferocytosis and slowing plaque progression, offering a synergistic therapeutic approach for atherosclerosis 75.

**Figure 4:**
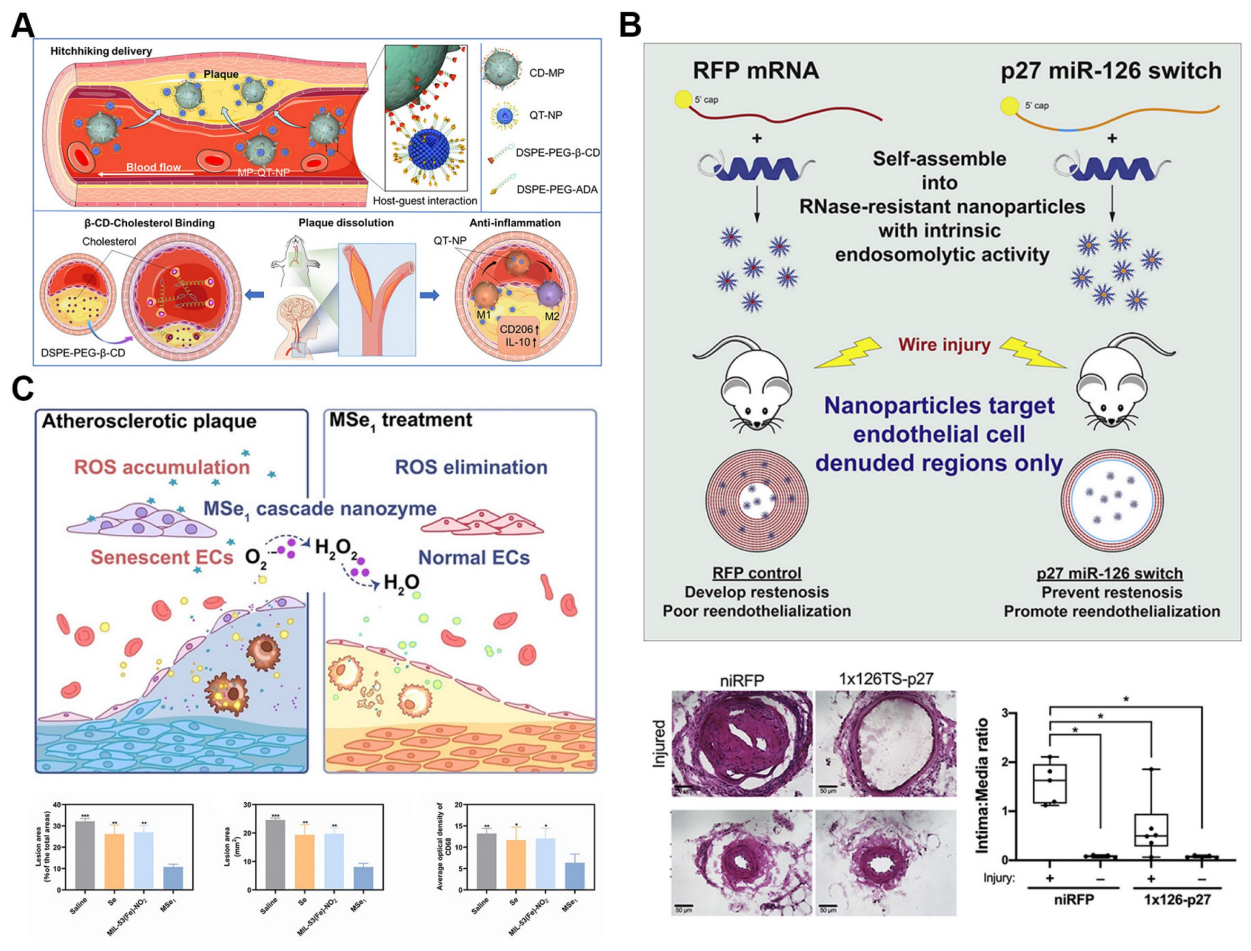
Self-assembled nanoparticles for atherosclerosis drug delivery. A. Schematic illustrations of the macrophage hithking MP-QT-NPs for atherosclerosis treatment. Reproduced with permission from 72. Copyright 2022, Elsevier. B. Illustration of the therapeutic mechanism and Effects of p27-miRNA switch-p5RHH Nanoparticles. The p27-miRNA switch-p5RHH nanoparticles selectively inhibit restenosis and facilitate vessel healing. Reproduced with permission from 73. Copyright 2021, Elsevier. C. Illustration of the therapeutic mechanism and therapeutic effects of MSe1 nanozyme for atherosclerosis. Reproduced with permission from 74. Copyright 2023, John Wiley and Sons.

In the realm of nanomedicine for atherosclerosis, cell hitchhiking strategies present significant advantages. By using natural cells, such as macrophages, as carriers, these strategies enable the efficient delivery of therapeutic agents directly to diseased sites. Mimicking immune cell behavior, cell-hitchhiking nanoparticles inherently target inflammatory regions, enhancing treatment specificity and efficacy. This approach also allows nanoparticles to evade immune clearance by camouflaging them with cell membranes, thereby extending their circulation time and improving therapeutic safety. A notable example of this strategy is the supramolecular macrophage-hitchhiking delivery system developed for anti-atherosclerosis therapy. Researchers designed nanoparticles (MP-QT-NPs) by attaching quercetin (QT)-loaded liposomes to the surfaces of macrophages using host-guest interactions mediated by β-cyclodextrin (β-CD) and adamantane (Figure 4A). These MP-QT-NPs efficiently accumulated in aortic lesions in atherosclerotic mice and significantly reduced the progression of atherosclerosis *in vivo* 72.

#### 5.1.2. Polymeric nanoparticles for atherosclerosis drug delivery

Polymeric nanoparticles have gained recognition as a promising drug delivery platform due to their unique physicochemical characteristics, including high biocompatibility, controlled release, and enhanced targeting abilities. These attributes make polymeric nanoparticles well-suited for addressing the complexities of atherosclerosis. Studies have shown that polymeric nanoparticles, such as PLGA nanoparticles, can restore lysosomal function by acidifying macrophages, thereby reducing plaque complexity and improving the pathological state of atherosclerosis 76. Recently, researchers developed an innovative amphiphilic low-molecular-weight heparin-unsaturated fatty acid conjugate (LMWH-uFA), designed to function both as an anti-atherosclerotic agent and a self-delivering nanocarrier. This conjugate self-assembles into micelles, with LMWH forming the outer shell and uFA composing the core, ensuring biological safety without the use of toxic additives. The hydrophilic LMWH segment inhibits early vascular inflammation by preventing monocyte adhesion, while the hydrophobic uFA segment regulates lipid levels. Moreover, by encapsulating RAP within the micelle core, its solubility was significantly enhanced, allowing targeted disruption of the P-selectin-mediated inflammatory cascade in the vasculature. These RAP-loaded nanoparticles not only reduced plaque size but also demonstrated the potential of this non-toxic nanocarrier to simultaneously target lipid regulation and inflammation *in vivo* 77. Despite their potential, prolonged use of polymeric nanoparticles, such as PLGA, may provoke inflammatory responses and exacerbate plaque progression. Thus, ensuring their long-term safety and efficacy in clinical applications remains a critical consideration 76.

#### 5.1.3. Peptide nanoparticles for atherosclerosis drug delivery

Peptide nanoparticles offer significant advantages for targeted atherosclerosis treatment due to their exceptional biocompatibility, customizable molecular design, effective functional modulation, and specific biological recognition capabilities 78, 79. Recent studies have demonstrated that a modified membrane-lytic peptide, melittin (p5RHH), can self-assemble into highly transfective, non-toxic nanoparticles when combined with synthetic mRNA. These nanoparticles possess intrinsic endosomolytic activity triggered by endosomal acidification (Figure 4B). In a femoral artery wire injury mouse model, the mRNA-p5RHH nanoparticles successfully delivered their therapeutic payload to areas of endothelial denudation, while sparing vital organs such as the lungs, liver, kidneys, and spleen. Notably, p5RHH nanoparticles loaded with synthetic mRNA encoding the cyclin-dependent kinase inhibitor p27Kip1—modified with an endothelial cell-specific miR-126 target sequence in the 5' UTR—significantly reduced neointimal hyperplasia and promoted re-endothelialization *in vivo*. This approach offers a cell-selective nanotherapy that provides targeted treatment for neointimal hyperplasia and atherosclerosis 73.

In another study, plaque-targeted selenopeptide nanoparticles were designed to precisely regulate the inflammatory immune microenvironment in atherosclerosis. These self-assembled selenopeptides integrate multiple bioactivities, including targeting, ROS-responsiveness, and therapeutic delivery, while maintaining high biocompatibility and minimal immunogenicity. Comprising a vascular adhesion molecule-1 (VCAM-1) targeting motif, an ROS-responsive seleno-amino acid linker, and a double-chained alkyl tail, the selenopeptide selectively accumulates in atherosclerotic tissues. Upon activation by ROS, the nanoparticles release anti-inflammatory drugs and generate bioactive seleno-metabolites, such as octadecyl selenite, which bind P-selectin with high affinity, inhibiting monocyte adhesion and macrophage-driven inflammation. This nanomedicine showed a 2.6-fold improvement in plaque inhibition compared to simvastatin *in vivo*, underscoring its potential as a safe and effective platform for inflammatory disease treatment 80.

#### 5.1.4. MOFs for atherosclerosis drug delivery

MOFs, known for their well-defined pore sizes, tunable composition, and versatile functionality, have emerged as promising drug delivery systems. Their nanoscale synthesis, adaptable surface chemistry, high loading capacity, and improved biocompatibility make them ideal for nanomedical applications 25. A novel anti-atherosclerosis treatment was developed by encapsulating losartan potassium (LP) within zeolitic imidazolate framework-8 (LP@ZIF-8) nanoparticles. This dual-therapy approach takes advantage of the enhanced permeability and retention (EPR) effect, confirmed by *in vivo* near-infrared fluorescence (NIRF) imaging. The ZIF-8 component promotes autophagy, helping regulate lipid metabolism and maintain cholesterol balance, while LP serves as an anti-inflammatory angiotensin receptor blocker (ARB). Together, these components demonstrate the therapeutic potential of LP@ZIF-8 for mitigating atherosclerosis severity 81. In another innovative study, MOFs were used to develop the multifunctional platform Rapa@UiO-66-NH-FAM-IL-1Ra (RUFI) for immunomodulatory therapy in atherosclerosis. RUFI integrates MOFs for delivering RAP and IL-1Ra, enabling immunomodulation and targeted therapy while incorporating 5-FAM for fluorescence imaging. *In vitro*, RUFI exhibited efficient drug release and selective cytotoxicity against inflammatory macrophages, and *in vivo*, it reduced plaque formation in atherosclerosis mouse models. This research highlights the potential of MOF-based co-delivery systems in enhancing immunoregulation for the treatment of atherosclerosis 82.

#### 5.1.5. Self-assembled nanozymes for atherosclerosis drug delivery

Self-assembled nanozymes have emerged as an innovative approach for treating atherosclerosis due to their enzyme-like activity and superior physicochemical properties compared to natural enzymes. Offering enhanced stability, durability, and cost-effectiveness, these nanozymes are well-suited for addressing the multifaceted pathophysiology of atherosclerosis, where oxidative stress, inflammation, and cellular senescence are key drivers of disease progression. Recent studies demonstrate that nanozymes not only function as therapeutic agents but also serve as multifunctional platforms, integrating drug delivery, imaging, and catalytic activity, thus amplifying their potential in atherosclerosis therapy 83.

Among these, MOF-based nanozymes have gained particular attention for their versatile applications in atherosclerosis treatment. With high loading capacity, controllable structures, and diverse catalytic functions, MOF-based nanozymes offer a robust platform for combined therapies. For example, MOF@Se nanozymes (MSe1) have shown efficacy in reducing cellular senescence and inflammation by scavenging excessive ROS in endothelial cells and macrophages (Figure 4C). These nanozymes not only protect DNA from oxidative damage but also inhibit foam cell formation, a critical process in plaque development. *In vivo* studies demonstrate that MSe1 nanozymes significantly slow atherosclerosis progression by mitigating oxidative stress and reducing inflammatory cell infiltration in plaques, showcasing their potential as a comprehensive therapeutic tool 74.

The multifunctionality of nanozymes is further highlighted by platelet membrane-coated biomimetic nanoplatforms (PCZ@PB NCs), which encapsulate the anti-atherosclerotic drug probucol. These platforms exploit the synergy between drug delivery and multienzyme activity to improve the oxidative and inflammatory microenvironment of atherosclerosis. By enhancing probucol's bioavailability and targeted delivery, these nanozymes reduce drug toxicity while maximizing therapeutic efficacy 84. Additionally, MOF-based nanozymes like DS-modified Cur/MOF@DS have exhibited excellent MRI capabilities and therapeutic potential by scavenging excessive ROS in plaque environments, further establishing the theranostic role of nanozymes in managing atherosclerosis 85.

#### 5.1.6. Self-assembled prodrug nanoparticles for atherosclerosis drug delivery

**Figure 5:**
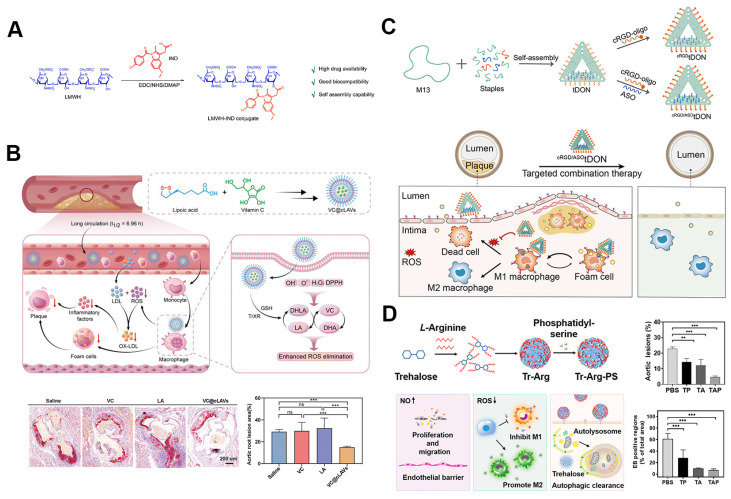
Self-assembled nanoparticles for atherosclerosis drug delivery. A. Structure of the self-assembled LMWH-IND prodrug nanoparticles for atherosclerosis treatment. Reproduced with permission 88. Copyright 2022, Elsevier. B. Illustrations of vitamin C encapsulating VC@cLAVs for treatment of atherosclerosis. VC@cLAVs enhanced the blood half-life of antioxidants and effectively reduced plaque area *in vivo*. Reproduced with permission 90. Copyright 2022, John Wiley and Sons. C. Illustration of DNA origami nanoparticle design and therapeutic mechanism for targeting M1 macrophages, reducing ROS, and mitigating foam cell formation in atherosclerotic plaques. Reproduced with permission from 91. Copyright 2024, American Chemical Society. D. Illustrations and therapeutic effects of carrier-free TAP nanomotors for atherosclerosis treatment. Reproduced with permission from 92. Copyright 2022, American Chemical Society.

Self-assembled drug delivery systems combining prodrug strategies with nanoscale technology hold significant promise in atherosclerosis treatment 86. One recent development is the functional nano-prodrug BUD-L-Arg@PSA designed to target activated endothelial cells via selective interaction with polysialic acid (PSA). This nano-prodrug combines budesonide (BUD), which exerts anti-inflammatory effects and upregulates eNOS expression, with l-arginine (L-Arg) to promote nitric oxide (NO) synthesis. By targeting atherosclerotic lesions, this system modulates the eNOS/NO and NF-κB inflammatory pathways, providing an effective alternative to surface modifications while maintaining targeted drug delivery 87. Another advancement involves self-assembled amphiphilic prodrugs, such as LMWH-IND (low-molecular-weight heparin-indomethacin conjugates), which specifically target P-selectin's role in plaque inflammation. Comprised of a hydrophilic low-molecular-weight heparin and a hydrophobic indomethacin component, the conjugate competitively binds to P-selectin, preventing the recruitment of mononuclear cells and macrophages, thereby inhibiting early vascular inflammation (Figure 5A). Indomethacin also stabilizes plaques by suppressing ROS and decreasing pro-inflammatory cytokine production from macrophages. This dual action results in reduced endothelial activation, significantly reducing plaque formation and alleviating inflammation *in vivo* 88.

##### 5.1.7. DNA origami and other SANPs for atherosclerosis drug delivery

In addition to the previously discussed self-assembled nanoparticles, several cutting-edge nanoparticle technologies are being developed for targeted atherosclerosis treatment. These innovations include DNA origami structures and carrier-free nanomotors, which offer improved targeting, stability, and therapeutic efficacy. Some biomaterials can self-assemble into nanoparticles, simultaneously carrying drugs while exhibiting inherent biological activity. This dual function enhances the biocompatibility and safety of nanomedicines, significantly amplifying their therapeutic effects. For example, β-CD, known for its strong interactions with hydrophobic cavities and cholesterol crystals (CCs), has been used in atherosclerosis treatment to improve methotrexate (MTX) loading and enhance cholesterol efflux. PEGylated β-CD loaded with MTX can self-assemble into nanoparticles (MTX NPs), which can be further modified with macrophage membranes (MM) to specifically target atherosclerotic plaques, increase cholesterol solubility, and suppress foam cell formation 89. Recently, Lu *et al.* developed VC@cLAVs, a novel nanodrug encapsulating vitamin C (VC) within lipoic acid-based cross-linked vesicles (cLAVs), which also act as natural antioxidants (Figure 5B). These vesicles are synthesized by cross-linking self-assembled lipoic acid in an aqueous solution, with VC in the hydrophilic core. The disulfide core and negatively charged surface of VC@cLAVs prevent blood dilution and protein adhesion. Once inside cells, VC@cLAVs dissociate into lipoic acid (LA) and dihydrolipoic acid (DHLA) and release both VC and its oxidized form, DHA. This design extends the antioxidant half-life by recycling the LA/DHLA and VC/DHA redox pairs. *In vivo*, VC@cLAVs reduced plaque area from 52% to 13%, significantly outperforming free VC (~45%) and LA (~38%) 90.

DNA origami technology has also gained significant attention as a novel nanoscale drug delivery system. Leveraging the self-assembly properties of DNA molecules, DNA origami constructs nanoscale structures with precise geometries and dimensions. This technique allows for the loading of various drug molecules and offers precise control over drug release. Furthermore, DNA origami provides excellent biocompatibility and degradability, reducing toxic side effects 93. A prime example of DNA origami's application in atherosclerosis treatment is the cRGD/ASOtDON DNA origami nanostructure (Figure 5C). This platform combines a cyclic RGD peptide targeting αvβ3 integrins, an antisense oligonucleotide (ASO) against miR-33 to enhance cholesterol efflux, and a DNA nanostructure designed to scavenge ROS. The nanostructure efficiently couples the cRGD peptide and loads the ASO with over 90% efficiency, enabling precise and effective gene delivery. The cRGD/ASOtDON nanostructure has demonstrated superior efficacy in reducing oxidative stress, reprogramming macrophages, and inhibiting foam cell formation—key factors in atherosclerosis progression—while achieving therapeutic effects at lower dosages compared to conventional drugs. Its high targeting specificity, low dosage requirement, and efficient systemic clearance minimize off-target effects and systemic immunosuppression, underscoring its potential as a safe and potent tool for atherosclerosis treatment 91.

Nanomotors represent a groundbreaking advancement that combines self-assembly and nanotechnology, offering autonomous movement for targeted drug delivery and modulation of pathological environments within the body. A notable example is the dual-mode nanomotor self-assembled from β-CD and LA, with immobilized gold nanoparticles. This nanomotor neutralizes ROS in inflamed areas, while β-CD aids in cholesterol removal from foam cells. The synergistic effect of these driving mechanisms enhances the nanomotor's ability to aggregate and penetrate plaques, thereby addressing the atherosclerotic microenvironment through combined therapies aimed at endothelial repair, lipid clearance, and ROS reduction 94.

In recent years, carrier-free nanomedicines, composed almost entirely of active pharmaceutical ingredients, have garnered attention for their high drug-loading capacity and minimized biosafety concerns. These systems also facilitate the co-delivery of multiple drugs through simple self-assembly, leading to improved therapeutic outcomes 47. Researchers have developed NO-driven carrier-free nanomotors, in which trehalose (Tr) and L-arginine self-assemble into nanoparticles (Tr-Arg), later modified with phosphatidylserine (PS) to form Tr-Arg-PS (TAP) nanomotors (Figure 5D). In the atherosclerotic environment, NO generated from Arg propels these nanomotors toward plaques. The PS coating enhances macrophage targeting, while ROS consumption regulates macrophage M2 polarization, and NO promotes endothelial barrier repair, providing a comprehensive therapeutic strategy. The TAP nanomotors demonstrated a 4.6-fold improvement in targeting efficiency, reducing the required dose of trehalose for autophagy induction, thus presenting a promising multi-targeted treatment for atherosclerosis 92.

Self-assembled nanoparticles have been extensively used in drug delivery for atherosclerosis, encompassing both carrier-mediated systems and nanoparticles that act as carriers. Table 4 summarizes their various types and applications in atherosclerosis treatment. These nanoplatforms enhance the targeting of therapeutic agents and gene therapies to diseased areas, effectively modulating inflammation.

### 5.2. Stimuli responsive self-assembled nanoparticles for atherosclerosis treatment

Despite their potential for drug delivery in atherosclerosis treatment, self-assembled nanoparticles face challenges such as nonspecific drug release during transit and inadequate drug release at the disease site, which significantly reduces therapeutic efficacy 43. To address these issues, stimuli-responsive nanosystems have recently emerged as a promising solution for targeted drug delivery 11, 45. These innovative designs focus on creating nanoparticles that respond to specific stimuli within the atherosclerotic microenvironment, including ROS, pH fluctuations, enzyme activity, and ATP levels. By leveraging these pathological conditions, self-assembled nanoparticles can achieve precise and controlled drug release directly at the disease site, thus improving therapeutic outcomes while minimizing systemic side effects.

#### 5.2.1. ROS responsive nanoparticles for atherosclerosis treatment

ROS play a fundamental role in the onset and progression of atherosclerosis by impairing endothelial function and promoting plaque formation. The significant increase in ROS levels within the atherosclerotic microenvironment has prompted the development of targeted ROS-responsive nanocarriers to specifically counteract these detrimental effects 96-98. For instance, ROS-responsive and size-reducible nanoassemblies have been designed using multivalent host-guest interactions between β-CD-anchored discoidal recombinant high-density lipoprotein (NP3ST) and hyaluronic acid-ferrocene (HA-Fc) conjugates. These HA-Fc/NP3ST nanoassemblies accumulate specifically in atherosclerotic plaques through the HA receptors CD44, which are overexpressed on injured endothelial cells. Upon exposure to elevated ROS levels in the intimal region, the nanoassemblies rapidly disassemble, releasing smaller NP3ST particles that penetrate deeper into the plaques. This enhances macrophage-targeted cholesterol efflux and drug delivery 99. Luo and colleagues developed another innovative ROS-sensitive carrier material, amphiphilic low molecular weight heparin-lipoic acid conjugate (LMWH-LA). This carrier, composed of clinically used injectable drug molecules, avoids potential unknown side effects. LMWH-LA, combined with curcumin (Cur), self-assembles into LLC nanoparticles, where LMWH forms the shell, and LA/Cur constitutes the core. In this system, LMWH targets P-selectin on plaque endothelial cells, blocking monocyte migration and thereby reducing ROS and inflammatory factor production. Simultaneously, the oxidation of LA triggers a hydrophilic-hydrophobic transformation, accelerating the release of therapeutic Cur. This approach effectively minimizes biotoxicity while maximizing therapeutic efficacy through stimuli-responsive nanoparticles 100.

**Figure 6:**
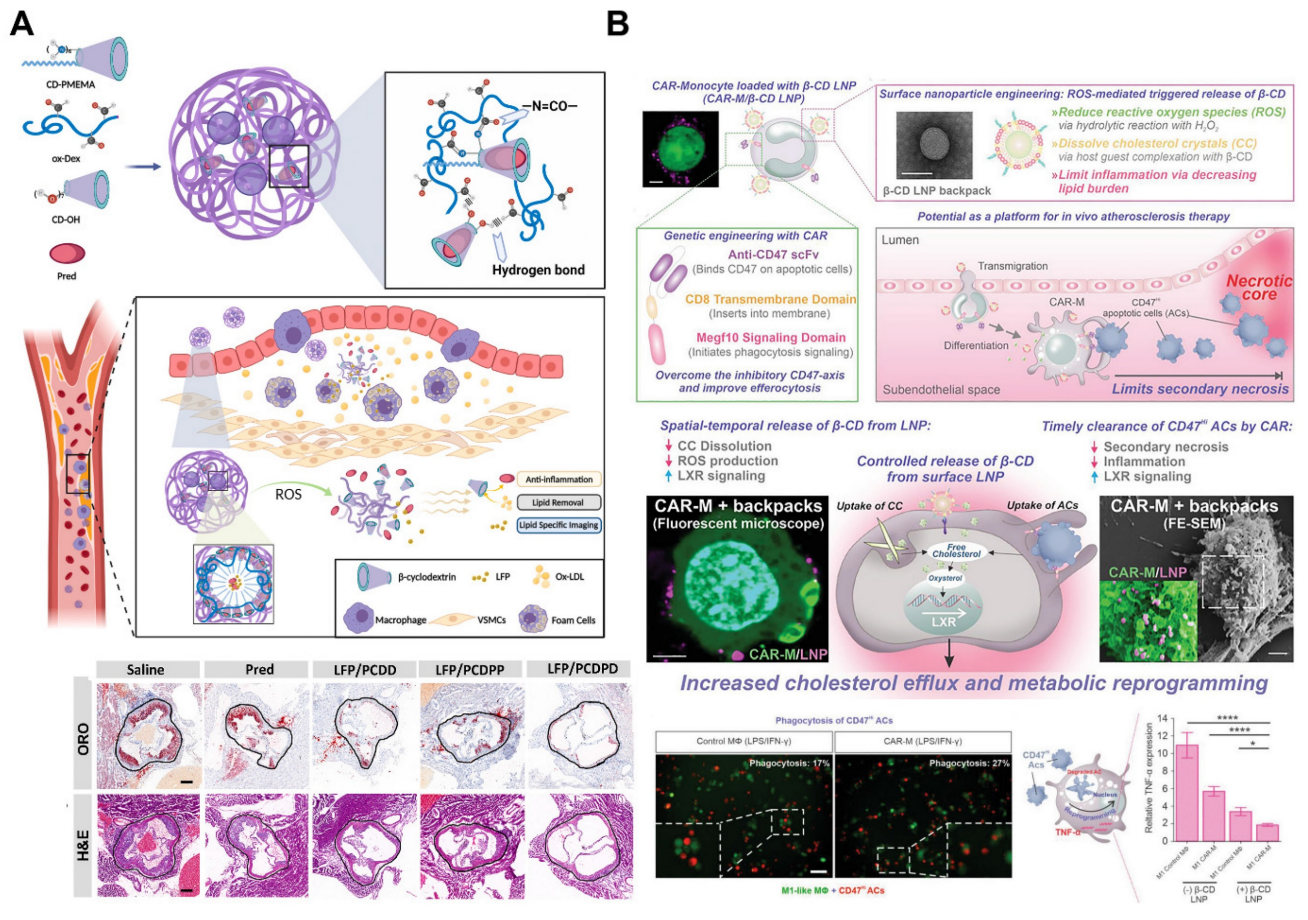
ROS responsive nanoparticles for atherosclerosis drug delivery. A. Schematic illustration of ROS responsive nanoparticles loaded with lipid-specific AIEgen and anti-inflammatory drug for targeted diagnosis and treatment of atherosclerosis. Reproduced with permission 101. Copyright 2022, Elsevier. B. Schematic illustration for the ROS responsive lipid nanoparticles-loaded CAR-Ms for potential atherosclerosis therapy. Reproduced with permission 103. Copyright 2024, John Wiley and Sons.

Similarly, the multifunctional ROS-responsive nanoparticle LFP/PCDPD has been developed, featuring a cyclodextrin structure that facilitates lipid removal and a PMEMA component that shifts from hydrophobic to hydrophilic upon ROS interaction. This nanoparticle not only exhibits anti-inflammatory and lipid-removing properties but also enhances active targeting to atherosclerotic sites due to the affinity of its dextran component for VCAM-1 and CD44 receptors on damaged endothelial cells. *In vivo*, LFP/PCDPD effectively reduces ROS levels, delivers the anti-inflammatory drug prednisolone (Pred), and removes lipids, achieving significant therapeutic results in atherosclerosis treatment (Figure 6A) 101. Recent advances have further integrated ROS responsiveness with diagnostic imaging and multifunctional therapies. For example, a novel nanoparticle platform combining ROS sensitivity with near-infrared-II photoacoustic imaging (NIR-II PAI) and siRNA therapy enables precise siRNA delivery to macrophages within plaques, downregulating pro-inflammatory pathways. This system also provides high-resolution imaging of ROS levels and the inflammatory microenvironment within plaques, offering a robust tool for monitoring therapeutic efficacy and assessing plaque vulnerability 102.

Another innovative approach combines therapeutic chimeric antigen receptor (CAR) macrophages with nanoparticle engineering to specifically target and eliminate apoptotic cells within atherosclerotic plaques. In this platform, the surface of CAR macrophages is modified with ROS-responsive nanoparticles that target the liver X receptor (LXR) pathway, enhancing their effector activities (Figure 6B). These β-CD LNPs release HPβ-CD under oxidative stress, dissolving cholesterol crystals and promoting oxygen sterol metabolism. Additionally, HPβ-CD upregulates the LXR pathway in macrophages, enhancing the clearance of apoptotic cell debris. CAR macrophages are specifically engineered to target and clear CD47-expressing apoptotic cells, which typically evade phagocytosis. This combination of CAR technology and nanoparticle engineering not only enhances lipid efflux but also increases the clearance rate of cell debris, thereby reducing inflammation within atherosclerotic plaques 103.

#### 5.2.2. pH responsive nanoparticles for atherosclerosis treatment

pH-responsive self-assembled nanoparticles exploit the pH differences between atherosclerotic sites and normal tissues to achieve targeted drug release. Chen *et al.* designed a pH-sensitive targeted nanoplex, LPLCH, to facilitate dual-track reverse cholesterol transport in atherosclerosis. The nanoplex's hyaluronic acid component specifically binds to overexpressed CD44 on foam cells in atherosclerotic sites, promoting the accumulation and internalization of LPLCH. This targeted delivery system undergoes pH-triggered charge conversion, enabling it to evade lysosomal degradation. The LXR agonist within the nanoparticle is then released, leading to the replacement of cholesterol esters (CE) and triggering LXR-mediated upregulation of ATP-binding cassette transporters A1/G1 (ABCA1/G1), which enhances local cholesterol efflux 104. Similarly, the pH-responsive, CD44-targeted nanoparticle H-CuS@DMSN-N C-HA was developed for the chemo-photo-thermal treatment of atherosclerosis. This system releases drugs in response to the acidic microenvironment of atherosclerotic plaques, facilitated by pH-sensitive Schiff base bonds. Both *in vitro* and *in vivo* studies have demonstrated the excellent biocompatibility and photothermal properties of H-CuS@DMSN-N C-HA, along with its ability to effectively target and ablate macrophages and thrombosis 105.

#### 5.2.3. Enzyme responsive nanoparticles for atherosclerosis treatment

The progression of atherosclerosis is closely linked to the abnormal activity of various enzymes, including hyaluronidase, matrix metalloproteinases (MMPs), and cathepsin K (CTSK). Overexpression of these enzymes at atherosclerotic lesion sites has prompted the development of enzyme-responsive nanoparticles. For example, secretory sphingomyelinase (SMase) plays a critical role in atherosclerosis progression by hydrolyzing sphingomyelin on the surface of LDL particles into ceramide. In response, researchers have developed nanomicelles containing iron oxide particles coated with sphingomyelin, which are selectively degraded by SMase, allowing the nanomicelles to accumulate within atherosclerotic plaques 106.

MMP-responsive nanogels have also been designed for atherosclerosis treatment, encapsulating the antioxidant and anti-atherosclerotic enzyme PON-1. Upon cleavage by MMP-2, PON-1 is released, reducing foam cell formation, LDL oxidation, and ROS levels 107. Additionally, a hyaluronidase (HAase)-sensitive drug delivery system has been synthesized by covalently attaching hyaluronic acid onto the surface of PLGA-reconstituted high-density lipoprotein (rHDL), termed HA-(C)-PLGA-rHDL. This HDL-mimetic nanocarrier targets infiltrating macrophages and enhances cholesterol efflux while delivering anti-atherosclerotic drugs 108.

**Figure 7:**
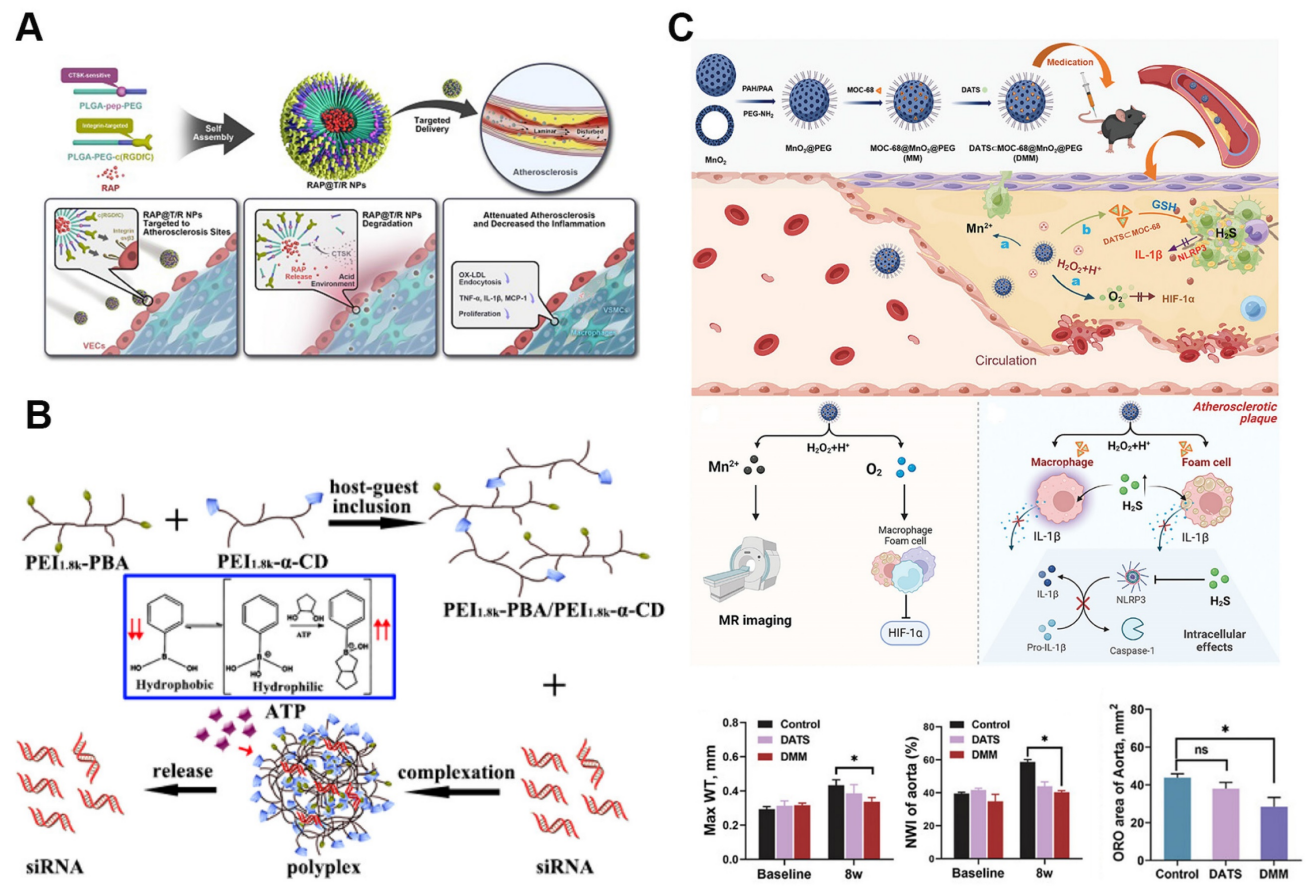
Various stimuli responsive nanoparticles for atherosclerosis treatment. A. Schematic illustration of cathepsin K (CTSK)-responsive RAP@T/R NPs targeting atherosclerosis. Reproduced with permission 109. Copyright 2022, Ivyspring International Publisher. B. Illustration of ATP-responsive polyplexes for gene delivery in atherosclerosis treatment. Reproduced with permission 110. Copyright 2019, American Chemical Society. C. Dual-stimuli-responsive DMM nanoparticles for atherosclerosis theranostics. This platform co-delivers H₂S and O₂ gases within plaques, reducing hypoxia and inflammation while enabling MR imaging. The MnO₂ component reacts with ROS and acidic pH within plaques, triggering gas release and anti-inflammatory effects. Reproduced with permission 111. Copyright 2024, John Wiley and Sons.

Among these enzymes, CTSK has emerged as a key target due to its elevated levels in atherosclerotic plaques. Nanoparticles responsive to CTSK, which target integrin αvβ3 and locally release RAP, are self-assembled from PLGA-PEG-c(RGDfC) and a CTSK-sensitive polymer, PLGA-Pep-PEG (Figure 7A). These RAP@T/R NPs are designed to accelerate RAP release in response to CTSK, reducing the phagocytosis of Ox-LDL and the secretion of inflammatory cytokines by macrophages. In addition to extending blood retention, these nanoparticles showed increased accumulation in atherosclerotic plaques and effectively prevented atherosclerosis progression and inflammation *in vivo* 109.

#### 5.2.4. ATP responsive nanoparticles for atherosclerosis treatment

ATP, a key cellular metabolite, plays a vital role in energy supply and intercellular signaling, with its concentration fluctuating across organelles, cellular compartments, and between healthy and diseased cells. These dynamic ATP levels have led to the development of ATP-responsive nanoparticles 112. In a recent study, an ATP-sensitive low-molecular-weight PEI-based supramolecular assembly was utilized for gene therapy in atherosclerosis. This assembly was synthesized via host-guest interactions between α-cyclodextrin (α-CD)-conjugated PEI and PEI 1.8k-phenylboronic acid (PBA) conjugates, enabling efficient intracellular disassembly and siRNA release (Figure 7B). The assembly exhibited excellent buffering capacity, protecting siRNA from RNase-mediated degradation, and *in vitro* tests confirmed its high cytocompatibility. LMW-PEI facilitated siRNA uptake via energy-dependent endocytosis. When combined with SR-A siRNA, the assembly effectively downregulated SR-A mRNA expression and inhibited the uptake of modified LDL 110. Additionally, a dual-targeting, multifunctional rHDL mimetic nanoplatform with an ATP-responsive trimeric core has been developed. This core includes an siRNA chip targeting SR-A and a catalase complex, while the phosphatidylserine-modified rHDL shell targets SR-BI and CD36, encapsulating pitavastatin. This ATP-responsive nanoparticle enhances plaque targeting through the mutual regulation of SR-A and CD36, significantly reducing plaque size and macrophage content *in vivo* 113.

#### 5.2.5. Dual stimuli responsive nanoparticles for atherosclerosis treatment

Dual- or multi-stimulus responsive nano-delivery platforms, by integrating multiple responsive elements, offer enhanced drug delivery efficiency compared to single-stimulus systems. These platforms not only improve therapeutic outcomes but also minimize systemic toxicity 82, 83. A prime example is the LAID nanoplatform, which responds to both oxidative stress and the acidic environment typical of atherosclerotic plaques. This platform is co-assembled from an iodinated contrast agent (ICA), boronated astaxanthin, and oxidized dextran (oxDEX), encapsulating a lipid-specific probe, LFP. The LAID nanoplatform specifically targets atherosclerotic sites through high-affinity CD44 interaction while combining X-CT and fluorescent imaging for early plaque detection. The binding of LFP to lipid cores facilitates the identification of vulnerable plaques, while astaxanthin treatment arrests plaque progression 114.

Additionally, diallyl trisulfide (DATS)-loaded MOC-68-doped MnO_2_ nanoparticles represent a microenvironment-responsive nanomedicine capable of co-delivering H_2_S and O_2_ to inflammatory cells within plaques. These nanoparticles display excellent monodispersity and stability, protecting DATS from degradation during circulation (Figure 7C). *In vitro*, this nanomedicine reduces macrophage polarization toward an inflammatory phenotype, inhibits foam cell formation, and suppresses the expression of NOD-like receptor pyrin domain-containing 3 (NLRP3) and interleukin-1β. *In vivo* studies demonstrate that it decreases plaque burden, reduces inflammatory infiltration, and alleviates hypoxia within plaques. Furthermore, therapeutic effects can be tracked in real time through the release of Mn^2+^ from the acidic and H_2_O_2_-responsive MnO_2_ nanoparticles 111. Another dual-stimulus responsive system involves simvastatin acid (SA)-loaded nanoparticles (SA PEI) bound to red blood cells (RBCs). This system facilitates high shear stress-responsive desorption of SA PEI from the RBC surface and ROS-responsive release of SA at atherosclerotic sites 115.

These recent advances emphasize the versatility of stimuli-responsive self-assembled nanoparticles in treating atherosclerosis. Table 5 summarizes key examples of such nanoparticles, their specific stimuli, therapeutic agents, and reported outcomes in treating atherosclerosis. These nano-delivery systems effectively target and deliver anti-inflammatory and lipid-reducing drugs to plaque sites, addressing inflammation modulation and lipid removal in atherosclerosis therapy.

### 5.3. SANPs for atherosclerosis vaccination

The development of atherosclerosis vaccines aims to overcome the limitations of conventional treatments, which often fail to effectively prevent disease progression 118. Self-assembling nanocarrier vaccines have shown significant potential in this area due to their capacity to precisely present specific antigens, thereby robustly stimulating immune responses against atherosclerosis. These vaccines facilitate the efficient delivery of both antigens and adjuvants to targeted immune cells, mimicking pathogen behavior and greatly enhancing the strength and longevity of the immune response 14. In atherosclerosis research, self-assembling nanocarriers—such as virus-like particles (VLPs), liposomes, micelles, and protein-based nanoparticles like ferritin—are used to develop vaccines that deliver antigens associated with the disease, including proprotein convertase subtilisin/kexin type 9 (PCSK9), apolipoprotein B (apoB), and S100A9.

**Figure 8:**
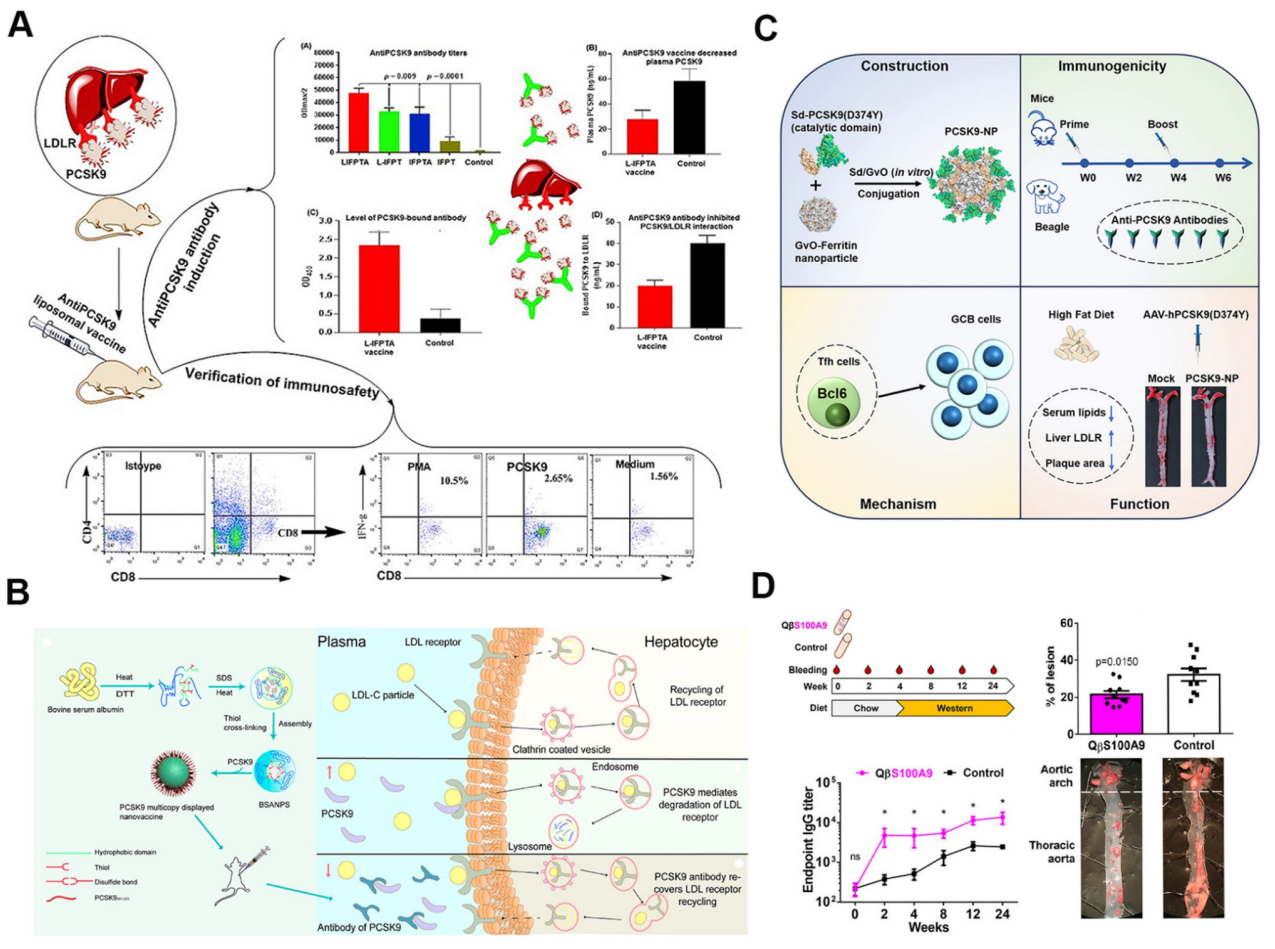
Self-assembled nanoparticles for atherosclerosis vaccine development. A. Mice inoculated with L-IFPTA vaccine showed higher serum antibody titers, suggesting the effectiveness of L-IFPTA. Reprinted with permission 122. Copyright 2019, Elsevier. B. Mechanism of PCSK9-targeted BSANPs (Bovine Serum Albumin Nanoparticles) in enhancing LDL receptor recycling and reducing PCSK9-mediated degradation, thereby improving cholesterol metabolism and reducing atherosclerotic plaque formation. Reproduced with permission from 124. Copyright 2024, American Chemical Society. C. Construction and immunogenicity of PCSK9-NP, including its conjugation with GvO-Ferritin nanoparticle, immunization regimen in animal models. Reproduced with permission 125. Copyright 2024, Elsevier. D. Vaccination of the QβS100A9 vaccine implant *in vivo* successfully achieved higher and stable level of S100A9-specific IgG, and the percentage of atherosclerotic lesion in the QβS100A9 vaccine implant group was lower than that in the control group. Reproduced with permission 126. Copyright 2022, John Wiley and Sons.

A key target for developing atherosclerosis nanovaccines is PCSK9, a critical regulator of lipid metabolism that facilitates the degradation of LDL receptors (LDLR) on hepatocyte surfaces 119. By eliciting a strong immune response against PCSK9, these nanovaccines prevent LDLR degradation, thereby enhancing receptor recycling and increasing availability. This mechanism leads to lower plasma LDL-C levels, reducing the formation and progression of atherosclerotic plaques. Studies have demonstrated that the immunogenic fused PCSK9-tetanus (IFPT) nanovaccine effectively lowers LDL cholesterol. The IFPT nanovaccine incorporates the PCSK9 peptide (a B-cell epitope) and a tetanus peptide (a T-cell epitope), conjugated via DSPE-PEG-maleimide on the liposome surface. When formulated with an alum adjuvant (L-IFPTA+), this nanovaccine induces the production of PCSK9-specific antibodies, lowering plasma PCSK9 levels, promoting LDLR recycling, and leading to a substantial reduction in LDL cholesterol (Figure 8A). Additionally, the IFPT nanovaccine has been shown to enhance anti-inflammatory immune responses, increasing CD4^+^ Th2 cells and IL-4 production while decreasing pro-inflammatory factors, thus contributing to the attenuation of atherosclerotic plaque development *in vivo* 120-122. Furthermore, another vaccine, PCSK9Qβ-003, utilizes VLPs as a carrier to target PCSK9. This vaccine alleviates atherosclerosis progression by promoting reverse cholesterol transport, reducing inflammatory infiltration, and inhibiting apoptosis. It has also demonstrated long-term and stable efficacy in lowering TC and LDL-C levels 123.

Self-assembling nanocarrier vaccines offer the added benefit of presenting multiple copies of antigens in a multivalent format, significantly enhancing immune responses and broadening immune activation. This capability improves the overall efficacy of vaccines. In one study, a PCSK9 multicopy display nanovaccine (PMCDN) was created by self-assembling bovine serum albumin (BSA) into sub-100 nm nanoparticles, followed by conjugating PCSK9 sequences onto the nanoparticle surface. This repetitive hapten display design produced a nanovaccine capable of inducing higher titers of PCSK9 antibodies, enhancing lymph node drainage, and improving antigen-presenting cell endocytosis compared to conventional PCSK9 peptide vaccines (Figure 8B) 124. Recently, Fang *et al.* developed an innovative PCSK9-targeted nanovaccine using ferritin as a nanocarrier. Ferritin, a naturally occurring iron-storage protein, is an excellent delivery platform due to its biocompatibility, ability to self-assemble into nanocage structures, and capacity for multivalent antigen display. In this study, the catalytic domain of PCSK9 (aa 153-aa 454, including the D374Y mutation) was covalently conjugated to self-assembled 24-mer ferritin nanoparticles, creating the PCSK9-NP vaccine (Figure 8C). This vaccine effectively induced neutralizing antibodies against PCSK9, significantly reduced blood lipid levels, and showed a remarkable reduction in aortic plaque lesions and macrophage infiltration *in vivo*. The inclusion of the D374Y mutation ensured the vaccine's efficacy in both normal and mutated PCSK9 populations 125.

Beyond PCSK9, other important targets for nanovaccines against atherosclerosis include apoB and P210. apoB is the primary structural protein of LDL and plays a key role in cholesterol transport and arterial plaque formation, contributing to atherosclerosis 118. Researchers developed liposomes encapsulating apoB100-derived peptides within DSPG (1,2-distearoyl-sn-glycero-3-phosphoglycerol), an anionic phospholipid. These DSPG-liposomes induced regulatory T cells (Tregs) specific to the encapsulated peptides, effectively reducing plaque formation by 50%, stabilizing plaques, and lowering serum cholesterol levels 127. P210, an immunogenic fragment of apoB100, has also emerged as a promising target for atherosclerosis treatment. Nanovaccines incorporating P210 into self-assembled micelles have shown significant efficacy in preclinical models by decreasing aortic plaque formation, regulating immune responses, and reducing the activation of pro-inflammatory and cytotoxic T cells 128.

S100A9 is an attractive target for nanovaccine development due to its critical role in atherosclerosis-related inflammation. It forms a heterodimer with S100A8, known as calprotectin, which is highly expressed in atherosclerotic plaques and contributes to leukocyte recruitment, cytokine secretion, and endothelial dysfunction. Targeting S100A9 with nanovaccines offers a promising strategy for reducing inflammation in atherosclerosis 129. A nanovaccine based on VLPs displaying S100A9 peptide epitopes, combined with slow-release PLGA implants, has been shown to effectively induce S100A9-specific antibodies (Figure 8D). This approach significantly lowered serum levels of calprotectin and pro-inflammatory cytokines, such as IL-1β, IL-6, and MCP-1, reducing inflammation and stabilizing plaques. Furthermore, the nanovaccine successfully diminished inflammatory cell infiltration in the arterial walls, significantly reducing the severity of aortic lesions and the risk of cardiovascular events. By directly modulating the inflammatory pathways involved in atherosclerosis, this nanovaccine presents a novel therapeutic strategy for preventing disease progression 126.

Self-assembled nanovaccines have the advantage of specifically targeting plaques and regulating immune responses, resulting in prolonged therapeutic effects and reduced inflammation. Additionally, their customizable design allows for personalized treatments, making them a versatile and effective approach to managing atherosclerosis. Table 6 highlights key examples of self-assembled nanoparticles used in developing atherosclerosis vaccines. However, further studies are necessary to thoroughly evaluate their safety and efficacy to ensure their clinical applicability.

### 5.4. SANPs for atherosclerosis theranostics

**Figure 9:**
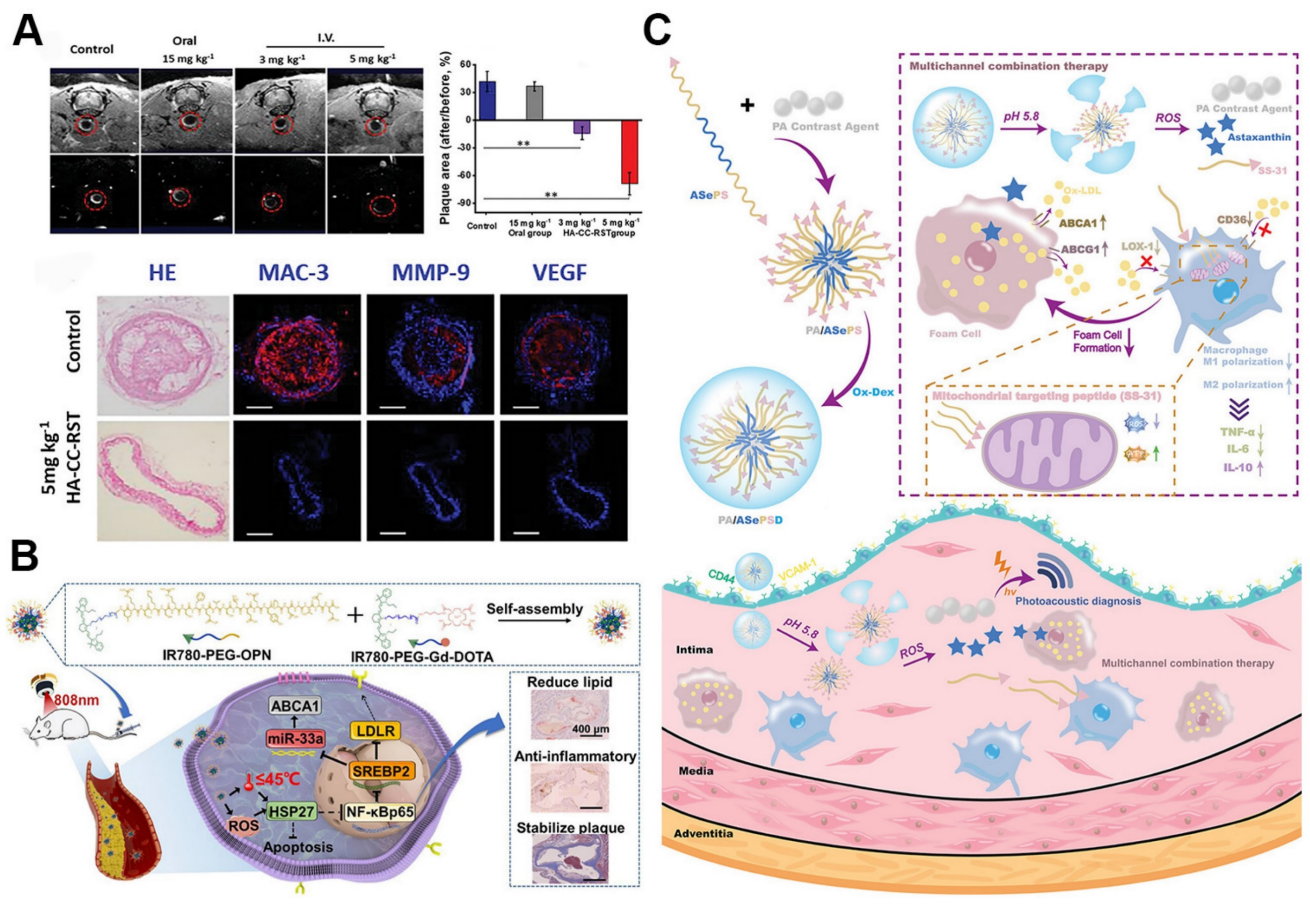
Self-assembled nanoparticles for atherosclerosis theranostics. A. MRI and histological analysis showing the reduction of plaque size and inflammation in mice treated with HA-CC-RST nanoparticles compared to controls. Reproduced with permission 133. Copyright 2022, John Wiley and Sons. B. Schematic illustration of the synthesis of IR780-Gd-OPN nanomicelles and the use of mild phototherapy to inhibit the progression of atherosclerotic plaques by enhancing the expression of heat shock protein 27, which subsequently inhibits the NF-κB pathway. Reproduced with permission 134. Copyright 2024, Elsevier. C. Illustration of pH and ROS-responsive PA/ASePSD nanoplatform targeting atherosclerotic plaques and mitochondria, enabling targeted photoacoustic diagnosis and combination therapy for atherosclerosis. Reproduced with permission 135. Copyright 2023, John Wiley and Sons.

Merging contrast and therapeutic agents into a unified theranostic nanoplatform for atherosclerosis offers a highly effective strategy for combining diagnostic, therapeutic, and monitoring functions, allowing for the concurrent detection, treatment, and management of the disease 130. This approach enables real-time tracking of disease progression, evaluation of therapeutic efficacy, and timely adjustments to treatment regimens, thereby facilitating personalized care 131, 132. Ma *et al.* developed hyaluronic acid-modified cerasome nano-agents (HA-CC) loaded with rosuvastatin (RST) and gadodiamide for targeted MRI imaging and atherosclerosis treatment (Figure 9A). Cerasomes (CCs), characterized by their silica-like surfaces, provide greater stability and biocompatibility than traditional liposomes. HA-CC, when loaded with gadodiamide, enhances MRI contrast in vulnerable plaques and significantly reduces plaque size. Transcriptomic analysis confirmed that HA-CC-RST markedly reverses atherosclerosis, with plaque area and volume reduced by 56.3% and 78.7%, respectively, at doses 50 times lower than oral RST 133.

Compared to theranostic platforms that rely on a single imaging modality, integrating self-assembled theranostic nanoplatforms with multimodal imaging overcomes inherent limitations such as low sensitivity and specificity, thereby significantly improving imaging resolution and diagnostic precision. One study introduced an innovative theranostic nanoplatform, MLNPs (9-CCN-[^125^I-ION/Cur]-LNPs), designed specifically for the targeted imaging and stabilization of vulnerable atherosclerotic plaques. These MLNPs accumulate in unstable plaques by incorporating "eat-me" signals (9-CCN) within their lipid membrane, ensuring efficient delivery of hybrid imaging agents ^125^I-ION and curcumin to macrophages. This precise targeting allows for accurate visualization of unstable plaques via SPECT and MRI, while simultaneously inducing M1-to-M2 macrophage polarization, contributing to plaque stabilization. This method provides a novel approach to the diagnosis and treatment of vulnerable plaques 136. Another study explored a different strategy for treating atherosclerosis by developing IR780-Gd-OPN nanomicelles, which combine mild phototherapy with advanced imaging capabilities (Figure 9B). These nanomicelles target foam cells within atherosclerotic plaques, using bone sialoprotein (OPN) for enhanced delivery and Gd-DOTA for MRI contrast. When activated by near-infrared light, the nanomicelles upregulate heat shock protein 27 (HSP27), inhibiting the NF-κB pathway, reducing lipid accumulation, promoting M2 macrophage polarization, and decreasing inflammation without inducing apoptosis. This approach not only improves plaque stability but also provides precise visualization of atherosclerosis treatment through high-sensitivity NIRFI and MRI 134.

More recently, researchers introduced the PA/ASePSD theranostic nanoplatform for multifunctional and intelligent treatment and diagnosis of atherosclerosis (Figure 9C). This nanoplatform integrates a π-conjugated polymer (PMeTPP-MBT) as a photoacoustic imaging agent with therapeutic components, such as the antioxidant SS-31 peptide and astaxanthin, which target mitochondria and regulate lipid metabolism. PA/ASePSD exhibits a unique response to the acidic, ROS-rich environment of atherosclerotic plaques, enabling controlled and targeted release of therapeutic agents that enhance anti-inflammatory effects, promote cholesterol efflux, and reduce cholesterol uptake by macrophages. As a theranostic platform, PA/ASePSD enables non-invasive, real-time photoacoustic imaging to detect early atherosclerotic lesions and, *in vivo*, effectively reduces plaque formation, inhibits macrophage infiltration, and repairs mitochondrial dysfunction 135.

In conclusion, the integration of diagnostic and therapeutic agents within self-assembled theranostic nanoplatforms represents a transformative breakthrough in atherosclerosis management. By enabling precise targeting, enhanced imaging, and effective treatment of plaques, these multifunctional nanoplatforms provide a comprehensive solution that not only improves diagnostic accuracy and therapeutic outcomes but also opens the door to personalized, real-time disease management. This innovation has the potential to revolutionize clinical practice and significantly improve patient care.

## 6. SANPs in biosensing for atherosclerosis

The integration of self-assembled nanomaterials with diverse analytical platforms, utilizing various sensing mechanisms, has led to the creation of nanosensors with enhanced attributes such as outstanding photostability, excellent biocompatibility, and high sensitivity 78, 137. In atherosclerosis, biological sensors are essential for early detection, monitoring disease progression, assessing risk, guiding treatment, evaluating drug efficacy, and analyzing physiological parameters. Among these, MOFs-based nanosensors stand out due to their unique properties. Self-assembled MOFs-based biosensors, characterized by their high surface area, tunable structures, porosity, and chemical stability, offer superior sensitivity, selectivity, rapid response, and customization, allowing for real-time monitoring of biological processes and the detection of trace biomolecules 138.

**Figure 10:**
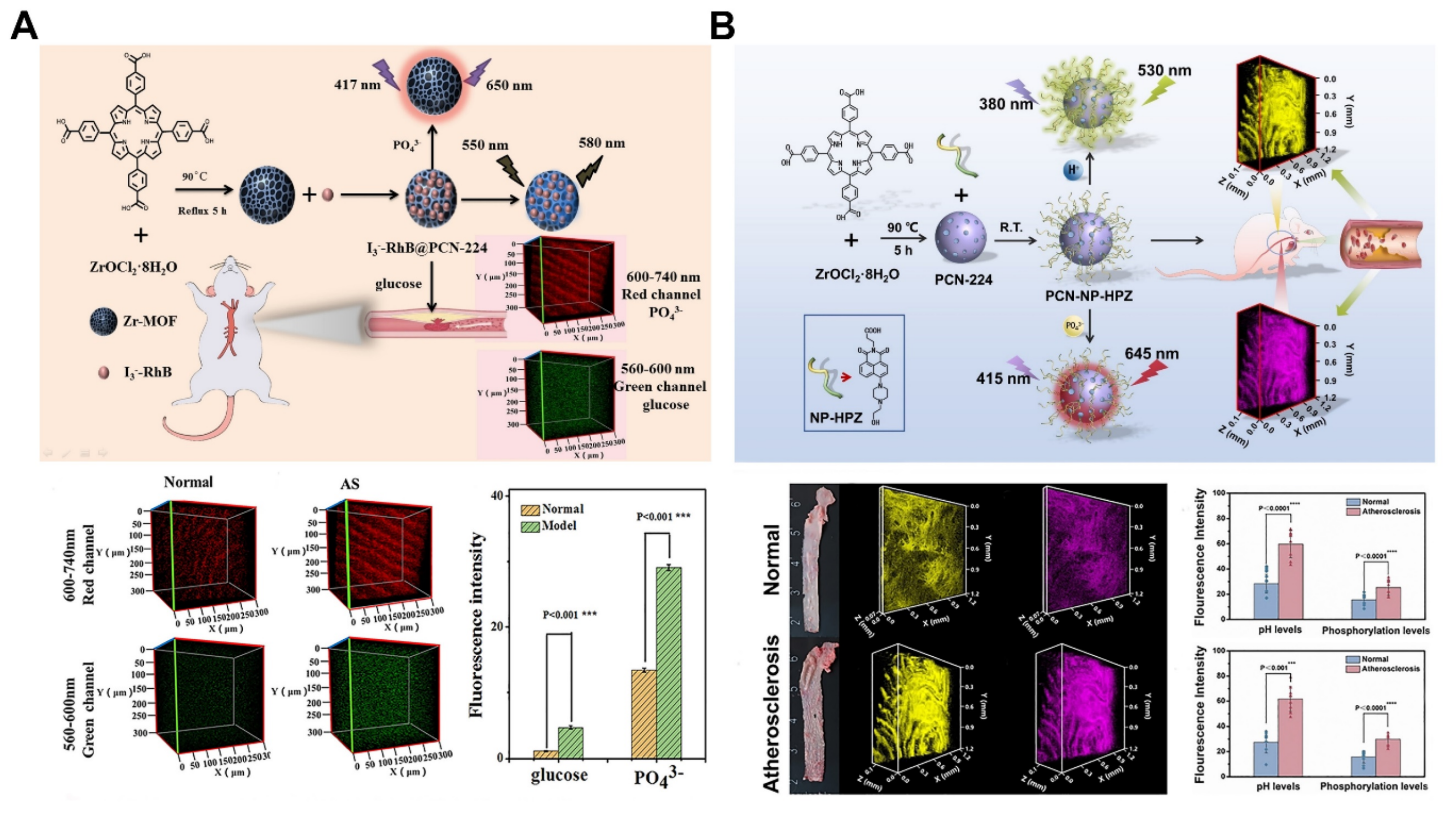
Self-assembled nanoparticles for atherosclerosis biosensing. A. Schematic illustration of synthesis of I_3_^-^-RhB @PCN-224 and its application in detection and imaging of phosphorylation and glucose levels in early atherosclerosis model. Two-photon imaging of the thoracic aorta showed early atherosclerosis mice displayed elevated levels of protein phosphorylation and glucose concentrations. Reproduced with permission 139. Copyright 2023, John Wiley and Sons. B. Schematic illustration of synthesis of PCN-NP-HPZ and its application for monitoring pH and phosphate levels in atherosclerosis development. Two-photon imaging of the aorta showed the increased levels of pH and phosphorylation in atherosclerosis mice. Reproduced with permission 140. Copyright 2022, John Wiley and Sons.

The inflammatory microenvironment, marked by variations in pH, protein phosphorylation, hypochlorous acid (HClO), and glucose levels, plays a critical role in the onset and progression of atherosclerosis. MOFs-based nanosensors provide a promising approach for real-time monitoring of these key parameters, offering insights into disease progression and enabling comprehensive condition assessments. For example, the MOFs-based fluorescent nanoprobe I_3_^-^-RhB@PCN-224 was designed for dual detection of glucose and protein phosphorylation, facilitating the evaluation of atherosclerosis by tracking levels in blood and tissue (Figure 10A). This study revealed elevated glucose and phosphate levels in the blood during early non-plaque stages of atherosclerosis compared to healthy mice. Two-photon imaging further demonstrated higher protein phosphorylation and glucose concentrations in early atherosclerosis models compared to healthy counterparts 139.

Additionally, the advanced nanoprobe PCN@FL was developed for simultaneous detection and imaging of phosphorylation and HClO in early atherosclerosis (Figure 10B). Selectivity for HClO was achieved through the specific interaction between dimethylthiocarbamate (DMTC) and HClO, while Zr(IV) facilitated phosphate detection. This probe successfully monitored fluctuations in HClO and phosphate levels in early-stage atherosclerosis mouse models, with two-photon microscopy highlighting increased levels of these markers on the aortic inner wall 141. Another dual-detection fluorescence nanosensor, PCN-NP-HPZ, was engineered through post-modification of MOFs to simultaneously monitor pH and phosphorylation. Incorporating a piperazine moiety and Zr(IV) nodes enhanced the sensor's dual functionality, enabling detailed monitoring of blood pH and phosphate levels during atherosclerosis development. Two-photon imaging demonstrated reduced pH and increased phosphorylation in the blood and tissue of atherosclerosis mice, illustrating the sensor's efficacy in early detection 140.

Furthermore, researchers explored the role of diabetes in triggering atherosclerosis by simulating blood circulation at the cellular level using mechanical stretching. A flexible glucose sensor (GOx@ZIF-8/Au SE) was developed by integrating glucose oxidase (GOx) encapsulated within a zeolitic imidazolate framework-8 (ZIF-8) with a stretchable gold electrode (Au SE). This design prevents GOx aggregation and detachment at the sensing interface, improving sensor stability. The stretchability of the Au SE enables both mechanical stimulation and real-time signal detection during mechano-transduction. The sensor revealed higher glucose utilization in static conditions compared to mechanically stretched cells under hyperglycemic conditions, offering insights into diabetes-induced atherosclerosis mechanisms and strategies for developing stable enzyme-based stretchable biosensors 142.

Looking ahead, SANPs-based biosensors are expected to revolutionize the detection and monitoring of atherosclerosis. Future advancements aim to enhance sensitivity and specificity, facilitate multi-marker analysis, and incorporate miniaturization and artificial intelligence. These innovations could lead to widespread clinical adoption, supporting personalized healthcare and improving disease prevention.

## 7. Clinical development

Self-assembled nanoparticles have not yet reached clinical application in atherosclerosis treatment, though their potential has been explored in various clinical trials, as outlined in Table 7. These trials primarily focus on HDL mimetic nanoparticles such as CER-001, CSL112, and MDCO-216, along with LN-PNP, PTX-LDE, and MTX-LDE. However, most clinical evaluations remain limited to simple nanoparticle formulations, lacking advanced or multifunctional technologies. Future trials should prioritize more sophisticated and multifunctional self-assembled nanodrugs to fully harness their therapeutic potential.

## 8. Conclusion and perspective

In recent years, self-assembled nanoparticles have garnered considerable attention for their capabilities in drug delivery across various diseases, including atherosclerosis. These nanoparticles offer distinct advantages, such as enhanced bioavailability, biodegradability, biocompatibility, and modifiability, which enable precise targeting and controlled release of therapeutic agents. These properties not only improve therapeutic outcomes but also minimize drug toxicity and adverse effects. Additionally, the straightforward production process of SANPs supports cost-effective, scalable manufacturing, thus accelerating their potential clinical translation.

Building on these attributes, SANPs have demonstrated significant promise in managing atherosclerosis, with a range of nanoparticle types developed, including lipid-based, protein-based, polymeric, peptide-based, MOFs, self-assembled nanozymes, and prodrug nanoparticles. Recently, DNA origami has also emerged as a novel strategy for targeted drug delivery in atherosclerosis, offering new possibilities for precision molecular therapies. These versatile nanoparticles can also carry imaging agents, enabling non-invasive imaging techniques such as MRI, photoacoustic imaging, and fluorescence imaging for early detection of atherosclerosis and identification of vulnerable plaques. Beyond diagnostic capabilities, SANPs can deliver therapeutic agents, bioactive compounds, and genetic materials directly to atherosclerotic sites, modulating inflammation and reducing plaque formation.

Self-assembling nanocarrier vaccines represent another promising avenue for the prevention and treatment of atherosclerosis. These nanocarriers deliver antigens and adjuvants to immune cells, effectively mimicking pathogenic behavior to stimulate a stronger immune response. By presenting multiple copies of antigens in a multivalent format, these vaccines significantly enhance immune activation and improve efficacy. The simultaneous delivery of imaging and therapeutic agents further facilitates the early diagnosis and timely treatment of vulnerable plaques. Emerging MOF-based biosensors are also anticipated to become key tools in the early diagnosis of atherosclerosis, providing personalized screening and management options.

The design of SANPs continues to evolve, with innovations increasingly focused on improving targeted and controlled delivery, as well as enhancing multifunctionality. Engineering SANPs through strategies such as active targeting, cell membrane coating, and cell hitchhiking has proven effective in refining targeting precision, optimizing drug distribution, and improving biocompatibility. Additionally, SANPs can be designed to respond to specific stimuli—such as acidic pH or enzymatic activity—triggering precise drug release, thereby minimizing systemic toxicity and maximizing therapeutic efficacy. With their high drug-loading capacity, precise targeting, and controlled release mechanisms, carrier-free SANPs are emerging as highly promising tools for treating atherosclerosis, significantly enhancing therapeutic outcomes while minimizing side effects.

The advent of microfluidic technology presents new opportunities for the scalable, cost-effective production of uniform, controllable self-assembled nanoparticles, which could accelerate the clinical translation of these nano-drugs. Moreover, innovative preparation strategies—such as *in vivo* self-assembling nanoparticles and self-assembling prodrug nanoparticles—are expected to further optimize the design and manufacturing processes of SANPs, pushing forward their clinical application.

Despite these advancements, several challenges hinder the clinical translation of SANPs. Many of the SANPs used in atherosclerosis research involve intricate modifications, and achieving rapid, precise, and reproducible synthesis remains a significant obstacle. Most research is still confined to *in vitro* studies and animal models, highlighting the need for further evaluation to confirm safety and efficacy in humans. Additionally, the immunostimulatory and immunosuppressive properties of SANPs present a unique set of challenges. Interactions with the immune system can sometimes provoke adverse responses, such as inflammation triggered by lipid or surfactant components, potentially compromising treatment effectiveness. Conversely, SANPs designed with membrane coatings or anti-inflammatory payloads may inadvertently suppress local immune responses, impairing the body's ability to combat infections. Therefore, comprehensive immune safety evaluations are critical for successful clinical translation, and extensive research is needed to ensure the safety and efficacy of SANPs in human applications.

## Figures and Tables

**Table 1:** Comparison of self-assembled nanoparticles and other nanoparticles

Aspects	Self-assembled nanoparticles	Other nanoparticles
Synthesis methods	Formed naturally through self-assembly under specific conditions	Typically synthesized via chemical processes or physical deposition
Structure	Highly ordered structure, self-organized	Structure may be uneven, depending on the synthesis method
Material properties	Highly controllable size and shape; superior physical and chemical stability	Properties may be less uniform
Surface functionalization	Can be optimized through self-assembly	May require additional chemical treatments
Cost	Can reduce production costs, especially at large scale	Higher production and functionalization costs
Environmental impact	Typically uses fewer harmful chemicals in the process	May involve more harmful chemicals
Preparation flexibility	Can be prepared under mild conditions, adaptable	Requires strict control conditions
Stability	Usually more stable in various environments	Stability may be limited by the synthesis process and storage conditions

**Table 2:** Comparison of self-assembled nanoparticles for atherosclerosis: characteristics, advantages, disadvantages, and applications

Types	Nanoparticles	Building blocks	Self-assembling driving force	Structure	Advantages	Disadvantages	Unique advantages and promising applications in atherosclerosis	Preparation methods	Refs.
Lipid based NPs	Liposome	Phospholipids, cholesterol	Hydrophobic interactions; electrostatic interactions	Lipid bilayer	•Biocompatibility •Biodegradability •Encapsulating both hydrophilic and hydrophobic drugs	•Potential for leakage of encapsulated drugs	Encapsulate hydrophilic and hydrophobic drugs, effective for plaque imaging and therapy	•Solvent evaporation •Solvent dispersion •Reverse phase evaporation •Ethanol injection followed by extrusion	48
	Lipid nanoparticles	Ionizable lipids, phospholipids, cholesterol	Hydrophobic interactions; electrostatic interactions	Lipid vesicles with homogeneous lipid core	•Biocompatibility •Biodegradability •Low immunogenicity •Ease of surface modification	•Poor loading capacity for hydrophilic drugs •Potential for drug leakage	Biocompatibility, targeted delivery to plaques, low immunogenicity, can be used for imaging	•Solvent evaporation •Ultrasound, high shear or pressure homogenization	18, 48, 49
Protein based NPs	HDL mimetic nanoparticles	Phospholipids, cholesterol, apolipoproteins or peptide mimics	Hydrophobic interactions; electrostatic interactions; van der Waals forces	HDL-like structures	•Mimic natural HDLs •Biocompatibility •Biodegradability •Ultrasmall size •Intrinsic targeting properties •Non-immunogenicity	•Complexity of synthesis •Stability concerns under physiological conditions	Mimics HDL, targets cholesterol efflux, ultra-small size, non-immunogenic	•Thermal cycling •Cholate dialysis •Sonication	50
	Ferritin	Ferritin subunits	Hydrophobic interactions; hydrogen bonding; electrostatic interactions	Spherical hollow nanocage	•Symmetrical spherical architecture •High thermal stability •Biocompatibility, •Biodegradability •Low toxicity	•Limited drug loading capacity •Potential immunogenicity	Spherical nanocage, delivers imaging and therapeutic agents, low toxicity	•Recombinant production using Escherichia coli	51
	Virus like particles (VLPs)	Viral proteins	Hydrophobic; interactions; electrostatic interactions	Virus-like structure	•Structural diversity; •Biocompatibility; •Bioactivity modulation; •Environmental friendliness.	•Potential immunogenicity	Modulates immune response, environmental friendliness, vaccine applications	•Prokaryotic/eukaryotic/cell-free expression systems	52
	Albumin	Albumin	Hydrophobic interactions; hydrogen bonding	Albumin	• Non-immunogenicity •Biodegradability •Long half-life •Excellent lyoprotectant for the solid form of nanomedicine	•Potential for aggregation •Variability in drug release profiles	Long circulation, non-immunogenic, delivers drugs and imaging agents, vaccine applications	•Desolvation •Emulsification •Thermal gelation •Nano-spray drying	53, 54
Peptide nanoparticles		Peptide sequences, functional groups	Hydrophobic interactions; hydrogen bonding	Variable structures (spherical, fibrous)	•Versatile functionality •Biodegradability •Targeted delivery potential	•Potential immunogenicity, •Complex synthesis and stabilization requirements	Versatile, targeted delivery to atherosclerosis-related proteins, diagnostics and therapy	•Self-assembly from aqueous solutions •Microfluidic assembly	21, 22
Polymeric nanoparticles		Polymer core, surfactants and stabilizers	Hydrophobic interactions; electrostatic interactions; van der Waals forces	Variable structures (core-shell, micelles)	•Controlled release •Biocompatibility •Tailored size and surface properties	•Potential toxicity from residual monomers or stabilizers •Immunogenicity	Controlled drug release, tailored size and surface properties, prolonged drug delivery	•Emulsion polymerization •Solvent evaporation •Nanoprecipitation	23, 24, 55
Metal orgainc frameworks		Metal ions or clusters, organic linkers	Coordination bonds; electrostatic interactions	Porous structure	•Large surface area; •Tailorable structure; •High porosity; •Tunable size; •Versatile functionality	•Stability concerns under physiological conditions •Potential cytotoxicity	Large surface area, high porosity, targeted drug delivery and imaging	•Hydro/solvothermal synthesis •Microwave-assisted synthesis •Electrochemical synthesis •Sonochemical synthesis	18, 25, 56
Self-assembled nanozymes		Enzymatic components	Various forces	Varies	•High catalytic activity •Biocompatibility	•Potential immunogenicity •Complex synthesis	High catalytic activity, ROS scavenging, anti-inflammatory role in atherosclerosis	•Hydro/solvothermal synthesis •Microwave-assisted synthesis •Electrochemical synthesis •Sonochemical synthesis	27, 28
Self-assembled prodrug nanoparticles		Prodrug molecules	Hydrophobic interactions; electrostatic interactions; van der Waals forces	Varies	•Direct delivery of active drugs without carriers	•Potential for premature drug release •Stability concerns	Direct drug delivery without carriers, localized therapy in plaques	•Solvent exchange •Nanoprecipitation •Self-assembly in aqueous solutions	17, 26
DNA origami		Single-stranded DNA scaffolds, staple strands	Hydrogen bonding; base pairing; electrostatic interactions	Precisely folded 2D or 3D DNA nanostructures	•High structural precision •Customizability and programmability •Biocompatibility and low toxicity •Capability to load and deliver multiple therapeutic and diagnostic agents	•Complex synthesis •Potential degradation in physiological conditions •High production costs	High structural precision, programmable, multi-targeting for drugs & imaging	•Self-assembly of complementary DNA strands	29, 30

HDL: high-density lipoprotein; NPs: nanoparticles; VLPs: virus-like particles

**Table 3:** Examples of developed self-assembled nanoparticles for non-invasive imaging of atherosclerosis.

Nanoplatform	Imaging agents	Imaging techniques	Advantages	Promising applications	Disadvantages	Refs.
LMWF	Gd-DTPA	MRI	Improved targeting specificity; enhanced relaxivity and signal intensity	Detection of activated HUVECs	Potential toxicity of Gadolinium-based contrast agents	58
Ratiometric semiconducting polymer nanoparticle	RSPN	PAI	High specificity, improved accuracy	Detection of oxidative stress and vulnerable plaques	May require optimization for deeper penetration	60
lipid-unlocking CTB-reactive probe	L-CRP	PAI	High signal specificity in lipid-rich environments; strong photoacoustic signal with deep tissue penetration	Diagnosis of plaques and risk stratification	Potential false activation in complex environments	61
MeOND	MeOND	FI	Strong near-infrared emission in low-polarity environments; high stability	Real-time imaging of LDs in plaques	Hydrophobicity might limit *in vivo* application	64
HA@PCFT	FC-TPA	FI	Dual-targeting capability; responsive fluorescence switching to improve imaging accuracy	Dual-targeting capability; responsive fluorescence switching to improve imaging accuracy; early diagnosis and assessment of plaque vulnerability	Specificity might vary with plaque composition	65
TPE-T-RCN	TPE-T-RCN	FI	High brightness and photostability in near-infrared range; AIE for enhanced contrast	precise early detection of plaques, rapid screening tool for evaluating anti-atherosclerosis drugs	May need further validation regarding stability and long-term safety	62
DCP liposomes	Cypate-PC	FI/PAI	Dual-functionality for fluorescence and photoacoustic imaging; specific uptake by foam cells	Photoacoustic and fluorescence imaging of atherosclerotic plaques	May increase foam cell accumulation	63
^89^Zr-radiolabeled liposomes	^89^Zr	PET/CT	High biodistribution visibility; long-term tracking of liposomes *in vivo*	Evaluating atherosclerotic burden, nanoparticle biodistribution	Potential radiation exposure concerns	68

AIE: aggregation-induced emission; CT: computed tomography; FI: fluorescence imaging; HUVECs: human umbilical vein endothelial cells; LD: lipid droplets; MRI: magnetic resonance imaging; PAI: photoacoustic imaging; PET: positron emission tomography

**Table 4:** Examples of self-assembled nanoparticles for drug delivery of atherosclerosis.

Types	Nanoplatforms	Delivering agents	Surface modifications	Targets	Results	Refs.
Lipid based nanoparticles	DHA liposomes	DHA	-	Atherosclerotic lesional macrophages	Promoted M2 macrophage polarization, reduced lipid deposition, increased collagen content, improved plaque stability	70
	AnxV-Rb1-LPs	Ginsenosides Rb1	AnxV	Phosphatidylserine in plaques	Removed cholesterol crystals, alleviated inflammation and apoptosis	71
	MM@Lips-SHP1i	SHP1i	Macrophage membranes	plaques	Promoted macrophage efferocytosis, reduced foam cell formation, inhibited pro-inflammatory cytokine expression, and achieved synergistic therapeutic effects	75
	MP-QT-NPs	Quercetin	β-cyclodextrin, adamantane	Aortic lesions	Reduced atherosclerosis, enhanced LXR activation and NRF2 expression, promoted cholesterol efflux	72
Polymeric nanoparticles	LMWH-uFA/RAP NPs	RAP, uFA	LMWH	P-selectin on activated ECs	Alleviated plaque inflammation, reduced plaque area	77
Peptide nanoparticles	p5RHH	siRNA, synthetic mRNA encoding p27Kip1	miR-126 target sequence	Endothelial cells, neointimal hyperplasia	Reduced neointimal hyperplasia, promoted re-endothelialization, high precision in targeted RNA delivery for AS treatment	73
	Selenopeptide nanoparticles	Anti-inflammatory drugs	ROS-responsive seleno-amino acid linker	VCAM-1, P-selectin, atherosclerotic lesions	Targeted drug delivery in response to ROS, reduced monocyte adhesion and macrophage inflammation, significantly reduced plaque formation and inflammation	80
Metal organic frameworks	LP@ZIF-8	Losartan potassium	IL-1Ra	IL-1RI on macrophages	Activated autophagy, exhibited anti-inflammatory effects	81
	RUFI	RAP, IL-1Ra, 5-FAM	VCAM-1-targeting	Atherosclerotic lesions	Modulated macrophage phenotype, reduced plaque formation	82
Self-assembled nanozymes	MSe1	Selenium	-	Endothelial cells, macrophages	Reduced cellular senescence and inflammation, protected DNA from oxidative damage, inhibited foam cell formation, reduced oxidative stress and inflammation in plaques	74
	PCZ@PB NCs	Probucol	Platelet membrane coating	Atherosclerotic plaques	Synergistic effects of drug delivery and multienzyme activity, reduced oxidative and inflammatory microenvironment, enhanced bioavailability, reduced drug toxicity	84
	DS-modified Cur/MOF@DS	Curcumin	DS	Atherosclerotic plaques	Scavenged excessive ROS in plaques, improved MRI performance, demonstrated theranostic potential in managing atherosclerosis	85
Self-assembled prodrug nanoparticles	BUD-L-Arg@PSA	BUD, L-Arginine	PSA	Activated endothelial cells	Modulates eNOS/NO and NF-κB pathways, upregulates eNOS, and promotes NO synthesis, providing targeted anti-inflammatory effects	87
	LMWH-IND	Indomethacin, LMWH	LMWH	P-selectin on activated endothelial cells	Targets plaque inflammation, prevents macrophage recruitment, reduces ROS and cytokine production, leading to significant plaque reduction.	88
Other self-assembled nanoparticles	β-CD/MTX nanoparticles	MTX	Macrophage membrane cloaking	Atherosclerotic lesions	Simplifies preparation, reduces toxicity, enhances bioavailability, and improves targeted delivery to lesions	89
	VC@cLAVs	VC	Lipoic acid -cross-linked vesicles	Atherosclerotic plaques	Prolongs half-life of antioxidants, enhances antioxidative capacity, and reduces plaque areas *in vivo*	90
	CIN@DEX5k-BSA/PTM/VB12	CIN	-	Gastrointestinal tract, atherosclerotic plaques	Enhances stability, increases oral bioavailability, and improves therapeutic outcomes against atherosclerosis	95
	cRGD/ASOtDON DNA origami	ASO against miR-33	Cyclic RGD peptide targeting αvβ3 integrins	Atherosclerotic plaques, αvβ3 integrins	Reduced oxidative stress, reprogrammed macrophages, inhibited foam cell formation, enhanced cholesterol efflux at lower dosages than conventional drugs	91
	β-CD/LA-Gold Nanomotor	β-cyclodextrin, L-arginine	Immobilized gold nanoparticles	Inflammatory zones, foam cells	Neutralizes ROS, removes cholesterol from foam cells, enhances aggregation and penetration within plaques, improves endothelial repair and lipid clearance	94
	NO-driven carrier-free nanomotor	Tr	PS	Atherosclerotic plaque microenvironment	Enhances Tr bioavailability and targeting efficiency, promotes macrophage autophagy, M2 polarization, endothelial barrier reconstruction, and plaque stabilization	92

AnxV: annexin V; ASO: antisense oligonucleotide; BUD: budesonide; CIN: cinnamaldehyde; DHA: docosahexaenoic acid; DS: disulfide; ECs: endothelial cells; MTX: methotrexate; NO: nitric oxide; PS: phosphatidylserine; PSA: polysialic acid; RAP: rapamycin; ROS: reactive oxygen species; SHP1i: SHP1 inhibitor; Tr: trehalose; uFA: unsaturated fatty acid; VC: vitamin C

**Table 5:** Examples of stimuli responsive self-assembled nanoparticles for atherosclerosis drug delivery.

Stimuli	Nanoparticles	Delivering agents	Results	Refs.
ROS	HA-Fc/NP^3^_ST_	Simvastatin	ROS responsive NP^3^ST release, reduced plaque size, plaque lipid deposition, plaque macrophage content and local inflammatory factor level	99
	LMWH-LA	Cur	Reduced monocyte migration, decreased ROS and inflammation, ROS-triggered release of Cur	100
	LFP/PCDPD	Lipid-specific AIE fluorescent probe, prednisolone	ROS response-mediated anti-inflammatory drug release, effective lipid specific of early atherosclerosis, synergistic treatment integrating anti-inflammation and lipid removal	101
	ROS-responsive si-Olfr2 NPs	siRNA (si-Olfr2)	Downregulated Olfr2, reduced inflammation, plaque formation, and necrotic areas. Enabled high-resolution NIR-II PAI for diagnosis	102
	β-CD LNPs	CAR macrophages, HPβ-CD	ROS responsive HPβ-CD release, promoted clearance of apoptotic cell resistant to phagocytosis and appoptic cell fragments	103
	RBC/LFP@PMMP	Fluorescent probe, prednisolone	ROS responsive release of Pred and LFP, accurate anti-inflammation and lipid-specific fluorescent imaging of atherosclerotic lesions	116
	KPF@MM-NPs	Kaempferol	Reduced inflammatory proliferation of macrophages, reduced key pro-inflammatory cytokines and re-polarization from M1 to M2 phenotypes	117
pH	LPLCH	Lver X receptor agonists	Inhibition on the plaque progression and a further reversal of formed lesions when under a healthy diet *in vivo*	104
	H-CuS@DMSN-N C-HA	Anticoagulant drug heparin, copper sulfide	pH responsive drug release and good photothermal properties of H-CuS@DMSN-N C-HA, ablation of macrophages and thrombosis *in vivo*	105
Enzyme (SMase)	Sphingomyelin iron oxide nanomicelles	Iron oxide particles	Destabilisation of SPHIONMs by SMase* in vitro*, accumulation of iron oxide-based nanomicelles in the plaque *in vivo*	106
Enzyme (MMP)	MMP-2 responsive nanogels	PON-1 enzyme	MMP triggered release of the PON-1 enzyme and its efficacy against the production of ox-LDL, reduction in macrophage foam cell and reactive oxygen species formation	107
Enzyme (hyaluronidase)	HA-(C)-PLGA-rHDL	Simvastatin	Enhanced cholesterol efflux, effectiveness of HA-(C)-PLGA-rHDL loaded with simvastatin	108
Enzyme (CTSK)	RAP@T/R NPs	RAP	Accelerated RAP release of RAP@T/R NPs in response to CTSK stimulation, blocking in atherosclerosis development and suppression in systemic and local inflammation	109
ATP	ATP-responsive LMW-PEI-based supramolecular assembly	SR-A siRNA	Facilitated cellular entry of siRNA via energy-dependent endocytosis by LMW-PEI-based supramolecular assembly; effectiveness of supramolecular assembly in knocking down SR-A mRNA and inhibiting uptake of modified LDL *in vitro*	110
	ATP-responsive rHDL mimetic nanoplatform	siRNA (SR-A), catalase, pitavastatin	Enhanced plaque targeting, reduced plaque area and macrophage content via regulation of SR-A and CD36	113
ROS/PH	LAID nanoparticles	LFP, ICA, AST	Dual responsive drug release, lipid specific imaging and the antioxidation and suppression of foam cell formation Identification of vulnerable plaque via fluorescent imaging, recognition of early-stage atherosclerotic plaques via X-CT, inhibition in progression of plaques	114
ROS/pH	DATS-loaded MOC-68-doped MnO₂ nanoparticles	DATS, MnO₂	Co-delivery of H₂S and O₂, reduced macrophage polarization, foam cell formation, inflammation, and plaque burden. Real-time MRI monitoring.	111
ROS/Shear stress	SA PEI@RBCs	SA	The high shear stress responsive desorption of SA PEI from the RBC surface, H_2_O_2_-responsive drug release of SA PEI@RBCs *in vitro*, reduced risk of SA bleeding, significantly improved therapeutic effects of atherosclerosis	115

AIE: aggregation-induced emission; AST: astaxanthin; CAR: chimeric antigen receptor; CTSK: cathepsin K; Cur: curcumin; DATS: diallyl trisulfide; ICA: iodinated contrast agent; LDL: low-density lipoprotein; LFP: lipid-specific probe; LMW-PEI: low-molecular-weight polyethylenimine; MMP: matrix metalloproteinase; Pred: prednisolone; RAP: rapamycin; ROS: reactive oxygen species; SA: simvastatin acid; SMase: sphingomyelinase; SR-A: scavenger receptor class A

**Table 6:** Examples of self-assembled nanoparticles for vaccination of atherosclerosis.

Vaccine	Type	Antigen Epitope	Vaccinated species	Refs.
Nanoliposomal IFTP vaccine	Liposmes	IFPT	Mouse, male rhesus macaque monkey	120-122
PCSK9Qβ-003 vaccine	VLPs	PCSK9-derived peptides	Mouse	123
PCSK9 multi-copy display nanovaccine	BSA nanoparticles	Sequences of PCSK9	Mouse and macaque	124
Ferritin-based PCSK9 nanovaccine	Ferritin	PCSK9 peptides	Mouse and beagle	125
DSPG-liposomes	Liposome	LDL-derived peptide antigen	Mouse	127
P210-PAMs	Micelles	P210 (ApoB-100)	Humanized mouse	128
Qβ VLP vaccine	VLPs	S100A9 peptide epitopes	Mouse	126

BSA: Bovine serum albumin; IFTP: immunogenic fused PCSK9-tetanus; LDL: low-density lipoprotein; PCSK9: proprotein convertase subtilisin/kexin type 9; VLPs: virus-like particles

**Table 7:** Summary of self-assembled nanoparticles used for atherosclerosis diagnosis and therapy in the clinical trials.

Nanomaterial	NPs	Year	Design	Outcomes	Refs.
HDL mimetic NPs	CER-001	2014	Phase 2, multicenter, randomized, double-blind, placebo-controlled	CER-001 infusions did not reduce coronary atherosclerosis.	143
	CER-001	2016	Single-center, observational	Increased plasma apoA-I concentration and plasma cholesterol efflux capacity	144
	CER-001	2017	Phase 2, multi-center, double-blind, placebo-controlled	CER-001 induced atheroma regression in patients with ACS.	145, 146
	CER-001	2020	Phase 3, multicenter, randomized, double blind, placebo controlled	CER-001 did not reduce atherosclerosis.	147
	CSL112	2015	Phase 2a, multi-center, randomized, double-blind, placebo-controlled	Elevated apoA-I levels and increased cholesterol	148
	CSL112	2018	Phase 1/phase 2a, randomized, double-blind, placebo-controlled, multicenter	Elevated cholesterol efflux in patients with low or high baseline HDL function.	149
	CSL112	2018	Phase 2a, randomized, double-blind, placebo-controlled, multicenter	CSL112 did not influence platelet aggregation.	150
	CSL112	2024	Phase 3, multicenter, double-blind, Randomized, placebo-controlled	CSL112 did not reduce the risk of myocardial infarction, stroke, or death from cardiovascular disease.	151
	MDCO-216	2016	Randomized, placebo-controlled	Increased cholesterol efflux, rapid changes in lipid levels and lipoprotein composition	152, 153
	MDCO-216	2018	Phase I, randomized, double-blind, placebo-controlled	MDCO-216 did not result in incremental plaque regression in the setting of contemporary statin therapy.	154
Liposomes	LN-PNP	2015	Randomized, placebo-controlled, double-blind	LN-PLP treatment did not reduce inflammation of arterial wall.	155
LDE	PTX-LDE	2016	/	Treatment with LDE-paclitaxel reduce atherosclerotic lesion size in patients.	156
	PTX-LDE	2020	Phase 2, randomized, double-blind, placebo-controlled	/	NCT04148833
	MTX-LDE	2020	Phase 2, randomized, double-blind, placebo-controlled	/	NCT04616872

ACS: acute coronary syndrome; LDE: lipid core nanoparticles; LN-PNP: liposomal nanoparticle encapsulating prednisolone; MTX: methotrexate; NPs: nanoparticles; PTX: paclitaxel

## Abbreviations

ABCA1/G1: ATP-binding cassette transporters A1/G1
ACS: acute coronary syndrome
AIE: aggregation-induced emission
AnxV: annexin V
ARB: angiotensin receptor blocker
ASO: antisense oligonucleotide
AST: astaxanthin
Au SE: gold stretchable electrode
BSA: bovine serum albumin
BUD: budesonide
CAR: chimeric antigen receptor
CAR-Ms: CAR macrophages
CCs: cerasomes
CD: cyclodextrin
CE: cholesterol esters
CIN: cinnamaldehyde
CT: computed tomography
CTSK: cathepsin K
Cur: curcumin
DATS: diallyl trisulfide
DHA: docosahexaenoic acid
DHLA: dihydrolipoic acid
DMTC: dimethylthiocarbamate
DOPS: dioleoylphosphatidylserine
DSPG: 1,2-distearoyl-sn-glycero-3-phosphoglycerol
DS: disulfide
ECs: endothelial cells
EPR: enhanced permeability and retention
FI: fluorescence imaging
Gox: glucose oxidase
HA: hyaluronic acid
Haase: hyaluronidase
HSP27: heat shock protein 27
HUVECs: human umbilical vein endothelial cells
ICA: iodinated contrast agent
IFPT: immunogenic fused PCSK9-tetanus
L-Arg: l-arginine
LA: lipoic acid
L-CRP: lipid-unlocking CTB-reactive probe
LDL: low-density lipoprotein
LDE: lipid core nanoparticles
LMWF: low molecular weight fucoidan
LMW-PEI: low-molecular-weight polyethylenimine
LN-PNP: liposomal nanoparticle encapsulating prednisolone
LNPs: lipid nanoparticles
LP: losartan potassium
LXR: liver X receptor
MMPs: matrix metalloproteinases
MM: macrophage membranes
MOFs: metal-organic frameworks
MRI: magnetic resonance imaging
MTX: methotrexate
NIR-II PAI: near-infrared-II photoacoustic imaging
NIRF: near-infrared fluorescence
NLRP3: NOD-like receptor pyrin domain-containing 3
NO: nitric oxide
NPs: nanoparticles
OPN: bone sialoprotein
PAI: photoacoustic imaging
PBA: phenylboronic acid
PCSK9: proprotein convertase subtilisin/kexin type 9
PET: positron emission tomography
PLGA: poly(lactic-co-glycolic acid)
PMCDN: PCSK9 multicopy display nanovaccine
Pred: prednisolone
PSA: polysialic acid
PS: phosphatidylserine
PTP: phosphatidylserine-targeting peptide
QT: quercetin
RAP: rapamycin
RBCs: red blood cells
ROS: reactive oxygen species
RSPNs: ratiometric photoacoustic semiconducting polymer nanoparticles
RST: rosuvastatin
SA: simvastatin acid
SANPs: self-assembled nanoparticles
SHP1i: SHP1 inhibitor
SMAse: sphingomyelinase
SMCs: smooth muscle cells
SNR: signal-to-noise ratio
Tr: trehalose
VC: vitamin C
VCAM-1: vascular adhesion molecule-1
VLPs: virus-like particles
ZIF-8: zeolitic imidazolate framework-8
oxDEX: oxidized dextran
ox-LDL: oxidized LDL
rHDL: reconstituted high-density lipoprotein
uFA: unsaturated fatty acid
O₂⁻: superoxide anions

## References

[1] Libby P, Buring JE, Badimon L, Hansson GK, Deanfield J, Bittencourt MS (2019). Atherosclerosis. Nat Rev Dis Primers.

[2] Hetherington I, Totary-Jain H (2022). Anti-atherosclerotic therapies: milestones, challenges, and emerging innovations. Mol Ther.

[3] Ma S, Xie X, Yuan R, Xin Q, Miao Y, Leng SX (2024). Vascular aging and atherosclerosis: a perspective on aging. Aging Dis.

[4] Dawson LP, Lum M, Nerleker N, Nicholls SJ, Layland J (2022). Coronary atherosclerotic plaque regression: JACC state-of-the-art review. J Am Coll Cardiol.

[5] Mushenkova NV, Summerhill VI, Zhang D, Romanenko EB, Grechko AV, Orekhov AN (2020). Current advances in the diagnostic imaging of atherosclerosis: insights into the pathophysiology of vulnerable plaque. Int J Mol Sci.

[6] Naghavi M, Ong KL, Aali A, Ababneh HS, Abate YH, Abbafati C (2024). Global burden of 288 causes of death and life expectancy decomposition in 204 countries and territories and 811 subnational locations, 1990-2021: a systematic analysis for the global burden of disease study 2021. Lancet.

[7] Newman CB, Preiss D, Tobert JA, Jacobson TA, Page RL 2nd, Goldstein LB (2019). Statin safety and associated adverse events: a scientific statement from the American Heart Association. Arterioscler Thromb Vasc Biol.

[8] Lee M, Cheng CY, Wu YL, Lee JD, Hsu CY, Ovbiagele B (2022). Association between intensity of low-density lipoprotein cholesterol reduction with statin-based therapies and secondary stroke prevention: a meta-analysis of randomized clinical trials. JAMA Neurol.

[9] Björkegren JLM, Lusis AJ (2022). Atherosclerosis: recent developments. Cell.

[10] Libby P (2021). The changing landscape of atherosclerosis. Nature.

[11] Liu Y, Jiang Z, Yang X, Wang Y, Yang B, Fu Q (2024). Engineering nanoplatforms for theranostics of atherosclerotic plaques. Adv Healthc Mater.

[12] Shi J, Xiao Z, Kamaly N, Farokhzad OC (2011). Self-assembled targeted nanoparticles: evolution of technologies and bench to bedside translation. Acc Chem Res.

[13] Zhang Y, Zhang Y, Ding R, Zhang K, Guo H, Lin Y (2024). Self-assembled nanocarrier delivery systems for bioactive compounds. Small.

[14] Vincent MP, Navidzadeh JO, Bobbala S, Scott EA (2022). Leveraging self-assembled nanobiomaterials for improved cancer immunotherapy. Cancer Cell.

[15] Li D, Zhang R, Liu G, Kang Y, Wu J (2020). Redox-responsive self-assembled nanoparticles for cancer therapy. Adv Healthc Mater.

[16] Cui W, Li J, Decher G (2016). Self-assembled smart nanocarriers for targeted drug delivery. Adv Mater.

[17] Nguyen A, Böttger R, Li SD (2021). Recent trends in bioresponsive linker technologies of prodrug-based self-assembling nanomaterials. Biomaterials.

[18] Xu Y, Fourniols T, Labrak Y, Préat V, Beloqui A, des Rieux A (2022). Surface modification of lipid-based nanoparticles. ACS Nano.

[19] Martínez-López AL, Pangua C, Reboredo C, Campión R, Morales-Gracia J, Irache JM (2020). Protein-based nanoparticles for drug delivery purposes. Int J Pharm.

[20] Kaltbeitzel J, Wich PR (2023). Protein-based nanoparticles: from drug delivery to imaging, nanocatalysis and protein therapy. Angew Chem Int Ed Engl.

[21] Negahdaripour M, Golkar N, Hajighahramani N, Kianpour S, Nezafat N, Ghasemi Y (2017). Harnessing self-assembled peptide nanoparticles in epitope vaccine design. Biotechnol Adv.

[22] Wickline SA, Hou KK, Pan H (2023). Peptide-based nanoparticles for systemic extrahepatic delivery of therapeutic nucleotides. Int J Mol Sci.

[23] Banik BL, Fattahi P, Brown JL (2016). Polymeric nanoparticles: the future of nanomedicine. Wiley Interdiscip Rev Nanomed Nanobiotechnol.

[24] Beach MA, Nayanathara U, Gao Y, Zhang C, Xiong Y, Wang Y (2024). Polymeric nanoparticles for drug delivery. Chem Rev.

[25] Ding M, Liu W, Gref R (2022). Nanoscale MOFs: from synthesis to drug delivery and theranostics applications. Adv Drug Deliv Rev.

[26] Wang W, Fan J, Zhu G, Wang J, Qian Y, Li H (2020). Targeted prodrug-based self-assembled nanoparticles for cancer therapy. Int J Nanomedicine.

[27] Zhang Y, Zhang C, Qian W, Lei F, Chen Z, Wu X (2024). Recent advances in MOF-based nanozymes: synthesis, activities, and bioapplications. Biosens Bioelectron.

[28] Wang D, Jana D, Zhao Y (2020). Metal-organic framework derived nanozymes in biomedicine. Acc Chem Res.

[29] Martynenko IV, Ruider V, Dass M, Liedl T, Nickels PC (2021). DNA origami meets bottom-up nanopatterning. ACS Nano.

[30] Hong F, Zhang F, Liu Y, Yan H (2017). DNA origami: scaffolds for creating higher order structures. Chem Rev.

[31] Zhang H, Zhu Y, Shen Y (2018). Microfluidics for cancer nanomedicine: from fabrication to evaluation. Small.

[32] Zhao Q, Cui H, Wang Y, Du X (2020). Microfluidic platforms toward rational material fabrication for biomedical applications. Small.

[33] Khoeini D, Scott TF, Neild A (2021). Microfluidic enhancement of self-assembly systems. Lab Chip.

[34] Sevim S, Sorrenti A, Franco C, Furukawa S, Pané S, deMello AJ (2018). Self-assembled materials and supramolecular chemistry within microfluidic environments: from common thermodynamic states to non-equilibrium structures. Chem Soc Rev.

[35] Maeki M, Uno S, Niwa A, Okada Y, Tokeshi M (2022). Microfluidic technologies and devices for lipid nanoparticle-based RNA delivery. J Control Release.

[36] Wang Y, Shim MS, Levinson NS, Sung HW, Xia Y (2014). Stimuli-responsive materials for controlled release of theranostic agents. Adv Funct Mater.

[37] Lee DE, Koo H, Sun IC, Ryu JH, Kim K, Kwon IC (2012). Multifunctional nanoparticles for multimodal imaging and theragnosis. Chem Soc Rev.

[38] Vaughan HJ, Green JJ, Tzeng SY (2020). Cancer-targeting nanoparticles for combinatorial nucleic acid delivery. Adv Mater.

[39] Sharma N, Bietar K, Stochaj U (2022). Targeting nanoparticles to malignant tumors. Biochim Biophys Acta Rev Cancer.

[40] Fang RH, Kroll AV, Gao W, Zhang L (2018). Cell membrane coating nanotechnology. Adv Mater.

[41] Liu Y, Luo J, Chen X, Liu W, Chen T (2019). Cell membrane coating technology: a promising strategy for biomedical applications. Nanomicro Lett.

[42] Wang Y, Sun SK, Liu Y, Zhang Z (2023). Advanced hitchhiking nanomaterials for biomedical applications. Theranostics.

[43] Krishnan N, Fang RH, Zhang L (2021). Engineering of stimuli-responsive self-assembled biomimetic nanoparticles. Adv Drug Deliv Rev.

[44] Zhang J, Lin Y, Lin Z, Wei Q, Qian J, Ruan R (2022). Stimuli-responsive nanoparticles for controlled drug delivery in synergistic cancer immunotherapy. Adv Sci (Weinh).

[45] Wei C, Jiang Z, Li C, Li P, Fu Q (2023). Nanomaterials responsive to endogenous biomarkers for cardiovascular disease theranostics. Adv Funct Mater.

[46] Liu Y, Li C, Yang X, Yang B, Fu Q (2024). Stimuli-responsive polymer-based nanosystems for cardiovascular disease theranostics. Biomater Sci.

[47] Fang F, Chen X (2024). Carrier-free nanodrugs: from bench to bedside. ACS Nano.

[48] Shah S, Dhawan V, Holm R, Nagarsenker MS, Perrie Y (2020). Liposomes: advancements and innovation in the manufacturing process. Adv Drug Deliv Rev.

[49] Eygeris Y, Gupta M, Kim J, Sahay G (2022). Chemistry of lipid nanoparticles for RNA delivery. Acc Chem Res.

[50] Chen J, Zhang X, Millican R, Creutzmann JE, Martin S, Jun HW (2020). High density lipoprotein mimicking nanoparticles for atherosclerosis. Nano Converg.

[51] Song N, Zhang J, Zhai J, Hong J, Yuan C, Liang M (2021). Ferritin: a multifunctional nanoplatform for biological detection, imaging diagnosis, and drug delivery. Acc Chem Res.

[52] Nooraei S, Bahrulolum H, Hoseini ZS, Katalani C, Hajizade A, Easton AJ (2021). Virus-like particles: preparation, immunogenicity and their roles as nanovaccines and drug nanocarriers. J Nanobiotechnology.

[53] Lamichhane S, Lee S (2020). Albumin nanoscience: homing nanotechnology enabling targeted drug delivery and therapy. Arch Pharm Res.

[54] An FF, Zhang XH (2017). Strategies for preparing albumin-based nanoparticles for multifunctional bioimaging and drug delivery. Theranostics.

[55] Elsabahy M, Wooley KL (2012). Design of polymeric nanoparticles for biomedical delivery applications. Chem Soc Rev.

[56] Kedar U, Phutane P, Shidhaye S, Kadam V (2010). Advances in polymeric micelles for drug delivery and tumor targeting. Nanomedicine.

[57] Jin R, Fu X, Pu Y, Fu S, Liang H, Yang L (2022). Clinical translational barriers against nanoparticle-based imaging agents. Adv Drug Deliv Rev.

[58] Cheng TM, Li R, Kao YJ, Hsu CH, Chu HL, Lu KY (2020). Synthesis and characterization of Gd-DTPA/fucoidan/peptide complex nanoparticle and in vitro magnetic resonance imaging of inflamed endothelial cells. Mater Sci Eng C Mater Biol Appl.

[59] Mallidi S, Luke GP, Emelianov S (2011). Photoacoustic imaging in cancer detection, diagnosis, and treatment guidance. Trends Biotechnol.

[60] Ma Y, Xu L, Yin B, Shang J, Chen F, Xu J (2021). Ratiometric semiconducting polymer nanoparticle for reliable photoacoustic imaging of pneumonia-induced vulnerable atherosclerotic plaque in vivo. Nano Lett.

[61] Ma Y, Shang J, Liu L, Li M, Xu X, Cao H (2023). Rational design of a double-locked photoacoustic probe for precise in vivo imaging of cathepsin B in atherosclerotic plaques. J Am Chem Soc.

[62] Wang K, Gao H, Zhang Y, Yan H, Si J, Mi X (2022). Highly bright AIE nanoparticles by regulating the substituent of rhodanine for precise early detection of atherosclerosis and drug screening. Adv Mater.

[63] Jiang YW, Tang WJ, Gao G, Geng YQ, Wu FG, Min Q (2022). Lipid droplet-hitchhiking probe creates Trojan foam cells for fluorescence/photoacoustic imaging of atherosclerotic plaques. Biosens Bioelectron.

[64] Liu Q, Li S, Ma D, Chen J, Li C, Zhuang W (2024). ROS-responsive nano-platform for lipid-specific fluorescence imaging of atherosclerosis. J Colloid Interface Sci.

[65] He Z, Chen Q, Duan X, Zhong Y, Zhu L, Mou N (2024). Reactive oxygen species-responsive nano-platform with dual-targeting and fluorescent lipid-specific imaging capabilities for the management of atherosclerotic plaques. Acta Biomater.

[66] Wang HP, Chen X, Qi YL, Huang LW, Wang CX, Ding D (2021). Aggregation-induced emission (AIE)-guided dynamic assembly for disease imaging and therapy. Adv Drug Deliv Rev.

[67] Lee SY, Jeon SI, Jung S, Chung IJ, Ahn CH (2014). Targeted multimodal imaging modalities. Adv Drug Deliv Rev.

[68] Lobatto ME, Binderup T, Robson PM, Giesen LFP, Calcagno C, Witjes J (2020). Multimodal positron emission tomography imaging to quantify uptake of (89)Zr-Labeled liposomes in the atherosclerotic vessel wall. Bioconjug Chem.

[69] Kiaie N, Gorabi AM, Penson PE, Watts G, Johnston TP, Banach M (2020). A new approach to the diagnosis and treatment of atherosclerosis: the era of the liposome. Drug Discov Today.

[70] Chong SY, Wang X, van Bloois L, Huang C, Syeda NS, Zhang S (2023). Injectable liposomal docosahexaenoic acid alleviates atherosclerosis progression and enhances plaque stability. J Control Release.

[71] Gong F, Wang Z, Mo R, Wang Y, Su J, Li X (2022). Nano-sponge-like liposomes remove cholesterol crystals for antiatherosclerosis. J Control Release.

[72] Gao C, Liu C, Chen Q, Wang Y, Kwong CHT, Wang Q (2022). Cyclodextrin-mediated conjugation of macrophage and liposomes for treatment of atherosclerosis. J Control Release.

[73] Lockhart JH, VanWye J, Banerjee R, Wickline SA, Pan H, Totary-Jain H (2021). Self-assembled miRNA-switch nanoparticles target denuded regions and prevent restenosis. Mol Ther.

[74] Liu W, Zhang Y, Wei G, Zhang M, Li T, Liu Q (2023). Integrated cascade nanozymes with antisenescence activities for atherosclerosis therapy. Angew Chem Int Ed Engl.

[75] Sha X, Dai Y, Chong L, Wei M, Xing M, Zhang C (2022). Pro-efferocytic macrophage membrane biomimetic nanoparticles for the synergistic treatment of atherosclerosis via competition effect. J Nanobiotechnology.

[76] Zhang X, Misra SK, Moitra P, Zhang X, Jeong SJ, Stitham J (2023). Use of acidic nanoparticles to rescue macrophage lysosomal dysfunction in atherosclerosis. Autophagy.

[77] Wang Q, Duan Y, Jing H, Wu Z, Tian Y, Gong K (2023). Inhibition of atherosclerosis progression by modular micelles. J Control Release.

[78] Zhou Y, Li Q, Wu Y, Li X, Zhou Y, Wang Z (2023). Molecularly stimuli-responsive self-assembled peptide nanoparticles for targeted imaging and therapy. ACS Nano.

[79] Qi GB, Gao YJ, Wang L, Wang H (2018). Self-assembled peptide-based nanomaterials for biomedical imaging and therapy. Adv Mater.

[80] Luo Z, Jiang Y, Liu Z, Guo L, Zhang L, Rong H (2024). Selenopeptide nanomedicine ameliorates atherosclerosis by reducing monocyte adhesions and inflammations. Nano Research.

[81] Sheng J, Zu Z, Zhang Y, Zhu H, Qi J, Zheng T (2022). Targeted therapy of atherosclerosis by zeolitic imidazolate framework-8 nanoparticles loaded with losartan potassium via simultaneous lipid-scavenging and anti-inflammation. J Mater Chem B.

[82] Xu Z, Wu Z, Huang S, Ye K, Jiang Y, Liu J (2023). A metal-organic framework-based immunomodulatory nanoplatform for anti-atherosclerosis treatment. J Control Release.

[83] Ren X, Chen D, Wang Y, Li H, Zhang Y, Chen H (2022). Nanozymes-recent development and biomedical applications. J Nanobiotechnology.

[84] Fu X, Yu X, Jiang J, Yang J, Chen L, Yang Z (2022). Small molecule-assisted assembly of multifunctional ceria nanozymes for synergistic treatment of atherosclerosis. Nat Commun.

[85] Lv F, Fang H, Huang L, Wang Q, Cao S, Zhao W (2024). Curcumin equipped nanozyme-like metal-organic framework platform for the targeted atherosclerosis treatment with lipid regulation and enhanced magnetic resonance imaging capability. Adv Sci (Weinh).

[86] Zhang Y, Cui H, Zhang R, Zhang H, Huang W (2021). Nanoparticulation of prodrug into medicines for cancer therapy. Adv Sci (Weinh).

[87] Wang S, Wang Y, Lai X, Sun J, Hu M, Chen M (2023). Minimalist nanocomplex with dual regulation of endothelial function and inflammation for targeted therapy of inflammatory vascular diseases. ACS Nano.

[88] Wang Q, Jing H, Lin J, Wu Z, Tian Y, Gong K (2022). Programmed prodrug breaking the feedback regulation of P-selectin in plaque inflammation for atherosclerotic therapy. Biomaterials.

[89] Zhu L, Li H, Li J, Zhong Y, Wu S, Yan M (2023). Biomimetic nanoparticles to enhance the reverse cholesterol transport for selectively inhibiting development into foam cell in atherosclerosis. J Nanobiotechnology.

[90] Lu X, He Z, Xiao X, Wei X, Song X, Zhang S (2023). Natural antioxidant-based nanodrug for atherosclerosis treatment. Small.

[91] Ma Y, Wang Q, Du S, Luo J, Sun X, Jia B (2024). Multipathway regulation for targeted atherosclerosis therapy using anti-miR-33-loaded DNA origami. ACS Nano.

[92] Wu Z, Zhou M, Tang X, Zeng J, Li Y, Sun Y (2022). Carrier-free trehalose-based nanomotors targeting macrophages in inflammatory plaque for treatment of atherosclerosis. ACS Nano.

[93] Jiang Q, Shang Y, Xie Y, Ding B (2024). DNA origami: from molecular folding art to drug delivery technology. Adv Mater.

[94] Wu Z, Wu R, Li X, Wang X, Tang X, Tan K (2022). Multi-pathway microenvironment regulation for atherosclerosis therapy based on beta-cyclodextrin/L-arginine/Au nanomotors with dual-mode propulsion. Small.

[95] Chen Y, Wang J, Xu J, Zhang J, Xu S, Zhang Q (2023). Fabrication of a polysaccharide-protein/protein complex stabilized oral nanoemulsion to facilitate the therapeutic effects of 1,8-cineole on atherosclerosis. ACS Nano.

[96] Shen M, Li H, Yao S, Wu X, Liu S, Yang Q (2021). Shear stress and ROS-responsive biomimetic micelles for atherosclerosis via ROS consumption. Mater Sci Eng C Mater Biol Appl.

[97] Tang D, Wang Y, Wijaya A, Liu B, Maruf A, Wang J (2021). ROS-responsive biomimetic nanoparticles for potential application in targeted anti-atherosclerosis. Regen Biomater.

[98] Yan R, Zhang X, Xu W, Li J, Sun Y, Cui S (2024). ROS-induced endothelial dysfunction in the pathogenesis of atherosclerosis. Aging Dis.

[99] He J, Zhang W, Zhou X, Xu F, Zou J, Zhang Q (2023). Reactive oxygen species (ROS)-responsive size-reducible nanoassemblies for deeper atherosclerotic plaque penetration and enhanced macrophage-targeted drug delivery. Bioact Mater.

[100] Luo X, Zhang M, Dai W, Xiao X, Li X, Zhu Y (2024). Targeted nanoparticles triggered by plaque microenvironment for atherosclerosis treatment through cascade effects of reactive oxygen species scavenging and anti-inflammation. J Nanobiotechnology.

[101] Xu H, She P, Ma B, Zhao Z, Li G, Wang Y (2022). ROS responsive nanoparticles loaded with lipid-specific AIEgen for atherosclerosis-targeted diagnosis and bifunctional therapy. Biomaterials.

[102] Ni H, Zhou H, Liang X, Ge Y, Chen H, Liu J (2024). Reactive oxygen species-responsive nanoparticle delivery of small interfering ribonucleic acid targeting olfactory receptor 2 for atherosclerosis theranostics. ACS Nano.

[103] Chuang ST, Stein JB, Nevins S, Kilic Bektas C, Choi HK, Ko WK (2024). Enhancing CAR macrophage efferocytosis via surface engineered lipid nanoparticles targeting LXR signaling. Adv Mater.

[104] Chen Z, Zhu Q, Li D, Lv Q, Fu G, Ma B (2024). Targeting nanoplatform for atherosclerosis inhibition and degradation via a dual-track reverse cholesterol transport strategy. Small.

[105] Liu S, Zhao Y, Shen M, Hao Y, Wu X, Yao Y (2022). Hyaluronic acid targeted and pH-responsive multifunctional nanoparticles for chemo-photothermal synergistic therapy of atherosclerosis. J Mater Chem B.

[106] Muñoz-Hernando M, Nogales P, Fernández-Barahona I, Ruiz-Cabello J, Bentzon JF, Herranz F (2024). Sphingomyelinase-responsive nanomicelles for targeting atherosclerosis. Nanoscale.

[107] Basak S, Khare HA, Roursgaard M, Kempen PJ, Lee JH, Bazban-Shotorbani S (2021). Simultaneous cross-linking and cross-polymerization of enzyme responsive polyethylene glycol nanogels in confined aqueous droplets for reduction of low-density lipoprotein oxidation. Biomacromolecules.

[108] Zhang M, He J, Jiang C, Zhang W, Yang Y, Wang Z (2017). Plaque-hyaluronidase-responsive high-density-lipoprotein-mimetic nanoparticles for multistage intimal-macrophage-targeted drug delivery and enhanced anti-atherosclerotic therapy. Int J Nanomedicine.

[109] Fang F, Ni Y, Yu H, Yin H, Yang F, Li C (2022). Inflammatory endothelium-targeted and cathepsin responsive nanoparticles are effective against atherosclerosis. Theranostics.

[110] Jiang C, Qi Z, Jia H, Huang Y, Wang Y, Zhang W (2019). ATP-responsive low-molecular-weight polyethylenimine-based supramolecular assembly via host-guest interaction for gene delivery. Biomacromolecules.

[111] Li D, Chen J, Lu Y, Yan X, Yang X, Zhang F (2024). Codelivery of dual gases with metal-organic supramolecular cage-based microenvironment-responsive nanomedicine for atherosclerosis therapy. Small.

[112] Deng J, Walther A (2020). ATP-responsive and ATP-fueled self-assembling systems and materials. Adv Mater.

[113] Jiang C, Qi Z, He W, Li Z, Tang Y, Wang Y (2019). Dynamically enhancing plaque targeting via a positive feedback loop using multifunctional biomimetic nanoparticles for plaque regression. J Control Release.

[114] Wang Y, Chen Z, Zhu Q, Chen Z, Fu G, Ma B (2024). Aiming at early-stage vulnerable plaques: A nanoplatform with dual-mode imaging and lipid-inflammation integrated regulation for atherosclerotic theranostics. Bioact Mater.

[115] Shen M, Jiang H, Zhao Y, Wu L, Yang H, Yao Y (2023). Shear stress and ROS dual-responsive RBC-hitchhiking nanoparticles for atherosclerosis therapy. ACS Appl Mater Interfaces.

[116] Ma B, Xu H, Wang Y, Yang L, Zhuang W, Li G (2021). Biomimetic-coated nanoplatform with lipid-specific imaging and ROS responsiveness for atherosclerosis-targeted theranostics. ACS Appl Mater Interfaces.

[117] Zhao J, Ling L, Zhu W, Ying T, Yu T, Sun M (2023). M1/M2 re-polarization of kaempferol biomimetic NPs in anti-inflammatory therapy of atherosclerosis. J Control Release.

[118] Shah PK, Chyu KY, Dimayuga PC, Nilsson J (2014). Vaccine for atherosclerosis. J Am Coll Cardiol.

[119] Shapiro MD, Tavori H, Fazio S (2018). PCSK9: from basic science discoveries to clinical trials. Circ Res.

[120] Momtazi-Borojeni AA, Jaafari MR, Banach M, Gorabi AM, Sahraei H, Sahebkar A (2021). Pre-clinical evaluation of the nanoliposomal antiPCSK9 vaccine in healthy non-human primates. Vaccines (Basel).

[121] Momtazi-Borojeni AA, Jaafari MR, Afshar M, Banach M, Sahebkar A (2021). PCSK9 immunization using nanoliposomes: preventive efficacy against hypercholesterolemia and atherosclerosis. Arch Med Sci.

[122] Momtazi-Borojeni AA, Jaafari MR, Badiee A, Sahebkar A (2019). Long-term generation of antiPCSK9 antibody using a nanoliposome-based vaccine delivery system. Atherosclerosis.

[123] Wu D, Pan Y, Yang S, Li C, Zhou Y, Wang Y (2021). PCSK9Qβ-003 vaccine attenuates atherosclerosis in apolipoprotein E-deficient Mice. Cardiovasc Drugs Ther.

[124] You S, Guo X, Xue X, Li Y, Dong H, Ji H (2019). PCSK9 hapten multicopy displayed onto carrier protein nanoparticle: an antiatherosclerosis vaccine. ACS Biomater Sci Eng.

[125] Fang Q, Lu X, Zhu Y, Lv X, Yu F, Ma X (2024). Development of a PCSK9-targeted nanoparticle vaccine to effectively decrease the hypercholesterolemia. Cell Rep Med.

[126] Ortega-Rivera OA, Shin MD, Moreno-Gonzalez MA, Pokorski JK, Steinmetz NF (2022). A single-dose Qβ VLP vaccine against S100A9 protein reduces atherosclerosis in a preclinical model. Adv Ther (Weinh).

[127] Benne N, van Duijn J, Lozano Vigario F, Leboux RJT, van Veelen P, Kuiper J (2018). Anionic 1,2-distearoyl-sn-glycero-3-phosphoglycerol (DSPG) liposomes induce antigen-specific regulatory T cells and prevent atherosclerosis in mice. J Control Release.

[128] Chyu KY, Zhao X, Zhou J, Dimayuga PC, Lio NW, Cercek B (2022). Immunization using apoB-100 peptide-linked nanoparticles reduces atherosclerosis. JCI Insight.

[129] Averill MM, Kerkhoff C, Bornfeldt KE (2012). S100A8 and S100A9 in cardiovascular biology and disease. Arterioscler Thromb Vasc Biol.

[130] Lammers T, Aime S, Hennink WE, Storm G, Kiessling F (2011). Theranostic nanomedicine. Acc Chem Res.

[131] Swierczewska M, Han HS, Kim K, Park JH, Lee S (2016). Polysaccharide-based nanoparticles for theranostic nanomedicine. Adv Drug Deliv Rev.

[132] Wang S, He H, Mao Y, Zhang Y, Gu N (2024). Advances in atherosclerosis theranostics harnessing iron oxide-based nanoparticles. Adv Sci (Weinh).

[133] Ma Q, Wu S, Yang L, Wei Y, He C, Wang W (2023). Hyaluronic acid-guided cerasome nano-agents for simultaneous imaging and treatment of advanced atherosclerosis. Adv Sci (Weinh).

[134] He W, Tu S, Han J, Cui H, Lai L, Ye Y (2024). Mild phototherapy mediated by IR780-Gd-OPN nanomicelles suppresses atherosclerotic plaque progression through the activation of the HSP27-regulated NF-κB pathway. Acta Biomater.

[135] Xu H, She P, Zhao Z, Ma B, Li G, Wang Y (2023). Duplex responsive nanoplatform with cascade targeting for atherosclerosis photoacoustic diagnosis and multichannel combination therapy. Adv Mater.

[136] Shi Z, Huang J, Chen C, Zhang X, Ma Z, Liu Q (2024). Lipid nanoparticles encapsulating curcumin for imaging and stabilization of vulnerable atherosclerotic plaques via phagocytic "eat-me" signals. J Control Release.

[137] Tang X, Zhu Y, Guan W, Zhou W, Wei P (2022). Advances in nanosensors for cardiovascular disease detection. Life Sci.

[138] Zhao Y, Zeng H, Zhu XW, Lu W, Li D (2021). Metal-organic frameworks as photoluminescent biosensing platforms: mechanisms and applications. Chem Soc Rev.

[139] Wen N, Li J, Zhang W, Li P, Yin X, Zhang W (2023). Monitoring the progression of early atherosclerosis using a fluorescence nanoprobe for the detection and imaging of phosphorylation and glucose Levels. Angew Chem Int Ed Engl.

[140] Li J, Zhao N, Zhang W, Li P, Yin X, Zhang W (2023). Assessing the progression of early atherosclerosis mice using a fluorescence nanosensor for the simultaneous detection and imaging of pH and phosphorylation. Angew Chem Int Ed Engl.

[141] Lu J, Li Z, Lu M, Fan N, Zhang W, Li P (2023). Assessing early atherosclerosis by detecting and imaging of hypochlorous acid and phosphorylation using fluorescence nanoprobe. Adv Mater.

[142] Peng M, Zhao X, Wang C, Guan L, Li K, Gu C (2021). In situ observation of glucose metabolism dynamics of endothelial cells in hyperglycemia with a stretchable biosensor: research tool for bridging diabetes and atherosclerosis. Anal Chem.

[143] Tardif JC, Ballantyne CM, Barter P, Dasseux JL, Fayad ZA, Guertin MC (2014). Effects of the high-density lipoprotein mimetic agent CER-001 on coronary atherosclerosis in patients with acute coronary syndromes: a randomized trial. Eur Heart J.

[144] Zheng KH, van der Valk FM, Smits LP, Sandberg M, Dasseux J-L, Baron R (2016). HDL mimetic CER-001 targets atherosclerotic plaques in patients. Atherosclerosis.

[145] Andrews J, Janssan A, Nguyen T, Pisaniello AD, Scherer DJ, Kastelein JJ (2017). Effect of serial infusions of reconstituted high-density lipoprotein (CER-001) on coronary atherosclerosis: rationale and design of the CARAT study. Cardiovasc Diagn Ther.

[146] Kataoka Y, Andrews J, Duong M, Nguyen T, Schwarz N, Fendler J (2017). Regression of coronary atherosclerosis with infusions of the high-density lipoprotein mimetic CER-001 in patients with more extensive plaque burden. Cardiovasc Diagn Ther.

[147] Zheng KH, Kaiser Y, van Olden CC, Santos RD, Dasseux J-L, Genest J (2020). No benefit of HDL mimetic CER-001 on carotid atherosclerosis in patients with genetically determined very low HDL levels. Atherosclerosis.

[148] Tricoci P, D'Andrea DM, Gurbel PA, Yao Z, Cuchel M, Winston B (2015). Infusion of reconstituted high-density lipoprotein, CSL112, in patients with atherosclerosis: safety and pharmacokinetic results from a phase 2a randomized clinical trial. J Am Heart Assoc.

[149] Gille A, D'Andrea D, Tortorici MA, Hartel G, Wright SD (2018). CSL112 (Apolipoprotein A-I [Human]) enhances cholesterol efflux similarly in healthy individuals and stable atherosclerotic disease patients. Arterioscler Thromb Vasc Biol.

[150] Gurbel PA, Tantry US, D'Andrea D, Chung T, Alexander JH, Bliden KP (2018). Evaluation of potential antiplatelet effects of CSL112 (Apolipoprotein A-I [Human]) in patients with atherosclerosis: results from a phase 2a study. J Thromb Thrombolysis.

[151] Gibson CM, Duffy D, Korjian S, Bahit MC, Chi G, Alexander JH (2024). Apolipoprotein A1 infusions and cardiovascular outcomes after acute myocardial infarction. N Engl J Med.

[152] Kallend DG, Reijers JA, Bellibas SE, Bobillier A, Kempen H, Burggraaf J (2016). A single infusion of MDCO-216 (ApoA-1 Milano/POPC) increases ABCA1-mediated cholesterol efflux and pre-beta 1 HDL in healthy volunteers and patients with stable coronary artery disease. Eur Heart J Cardiovasc Pharmacother.

[153] Kempen HJ, Gomaraschi M, Simonelli S, Calabresi L, Moerland M, Otvos J (2016). Persistent changes in lipoprotein lipids after a single infusion of ascending doses of MDCO-216 (apoA-IMilano/POPC) in healthy volunteers and stable coronary artery disease patients. Atherosclerosis.

[154] Nicholls SJ, Puri R, Ballantyne CM, Jukema JW, Kastelein JJP, Koenig W (2018). Effect of infusion of high-density lipoprotein mimetic containing recombinant apolipoprotein A-I Milano on coronary disease in patients with an acute coronary syndrome in the MILANO-PILOT trial: a randomized clinical trial. JAMA Cardiol.

[155] van der Valk FM, van Wijk DF, Lobatto ME, Verberne HJ, Storm G, Willems MC (2015). Prednisolone-containing liposomes accumulate in human atherosclerotic macrophages upon intravenous administration. Nanomedicine.

[156] Shiozaki AA, Senra T, Morikawa AT, Deus DF, Paladino-Filho AT, Pinto IM (2016). Treatment of patients with aortic atherosclerotic disease with paclitaxel-associated lipid nanoparticles. Clinics (Sao Paulo).

